# Metastable Crystalline Cobalt Iron Oxide Nano‐Flakes with Antiferromagnetic/Ferrimagnetic Composition Mosaicity

**DOI:** 10.1002/anie.202504171

**Published:** 2025-10-21

**Authors:** Anna Rabe, Franz‐Philipp Schmidt, Shohreh Rafiezadeh, Soma Salamon, Joachim Landers, Mirco Eckhardt, Christoph Pratsch, Benedikt Beckmann, Felix Thomas Haase, David Kordus, Mauricio Lopez Luna, Clara Rettenmaier, Thomas Götsch, Axel Knop‐Gericke, Arno Bergmann, Janis Timoshenko, Beatriz Roldan Cuenya, Oliver Gutfleisch, Mirijam Zobel, Rossitza Pentcheva, Heiko Wende, Thomas Lunkenbein, Malte Behrens

**Affiliations:** ^1^ Faculty of Chemistry University of Duisburg‐Essen Universitätsstr. 7 45141 Essen Germany; ^2^ Faculty of Physics University of Duisburg‐Essen and Center for Nanointegration Duisburg‐Essen (CENIDE) Lotharstr. 1 47057 Duisburg Germany; ^3^ Department of Inorganic Chemistry Fritz Haber Institute of the Max Planck Society 14195 Berlin Germany; ^4^ Department of Chemistry University of Bayreuth Universitätsstr. 30 95440 Bayreuth Germany; ^5^ Department of X‐ray Microscopy Helmholtz‐Zentrum Berlin für Materialien und Energie GmbH Albert‐Einstein‐Str. 15 12489 Berlin Germany; ^6^ Institute of Materials Science Technische Universität Darmstadt 64287 Darmstadt Germany; ^7^ Institute of Crystallography RWTH Aachen University 52066 Aachen Germany; ^8^ Department of Interface Science Fritz Haber Institute of the Max Planck Society Faradayweg 4‐6 14195 Berlin Germany; ^9^ Department of Heterogeneous Reactions Max Planck Institute for Chemical Energy Conversion Stiftstraße 34‐36 45470 Mülheim an der Ruhr Germany; ^10^ Present address: Bavarian Center for Battery Technology and Chair of Operando-Analytics for Electrochemical Energy Storage University of Bayreuth Universitätsstraße 30 95447 Bayreuth Germany; ^11^ Institute of Inorganic Chemistry and Kiel Nano Interface & Surface Science (KiNSIS) Kiel University May‐Eyth‐Str. 2 24118 Kiel Germany

**Keywords:** Exchange bias, Magnetic properties, Phase diagrams, Spinel phases, Synthetic methods

## Abstract

By thermal decomposition of a crystalline hydroxycarbonate precursor with a Co:Fe ratio of 2:1, crystals with alternating ferrimagnetic and antiferromagnetic nano‐domains were synthesized using a facile synthetic approach that combined bottom‐up co‐precipitation of the precursor with a self‐assembled top‐down nano‐structuring during spinel formation. Due to the miscibility gap of the spinel phase diagram at this composition, a topotactic segregation into CoFe_2_O_4_‐like and Co_3_O_4_‐like domains takes place at 400 °C, giving rise to porous crystalline nano‐flakes with spatial compositional fluctuations on a scale of approximately 5 nm. Experimental methods and density functional theory showed that the metastable nature of this interface‐rich material is manifested in the unexpectedly low lattice parameter of the iron‐rich domains, which can be explained by the compressive strain executed on this phase due to mosaicity. Investigations of the magnetic properties revealed an exchange bias effect, due to this unique microstructure, which is typically known for thin films or core/shell nanoparticles. Treatment at temperatures higher than 450 °C causes this microstructure to break down, the lattice strain to relax, and finally leads to properties expected for the thermodynamically stable phases according to the phase diagram.

## Graphical abstract

Through precursor co‐precipitation and mild calcination, a metastable interface‐rich cobalt iron oxide with unique magnetic properties was synthesized. Due to compression of the lattice parameter in the iron‐rich phase and the multitude of interfaces, an exchange bias was created, usually not known for such powder samples.

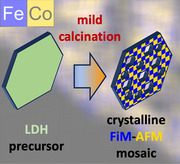

## Introduction

Spinel type oxides, especially those containing iron and/or cobalt, are multi‐functional and low‐cost materials in a broad variety of applications and their production via multiple routes in addition to their compositional flexibility, allows fine tuning of the desired properties.^[^ ^1^, ^2^, ^3^, ^4^ ^]^ However, in powder materials, one usually investigates and tunes the properties of uniform single phase spinels, while composition gradients in thin films^[^ ^5^, ^6^, ^7^ ^]^ have shown great potential to enhance the accessible range of properties. Here we present a novel and cost‐effective synthetic approach to spinel powders consisting of crystalline nanoparticles with mosaic composition fluctuations which exhibit noteworthy structural and magnetic properties observed for the first time in this kind of powder sample.

**Figure 1:**
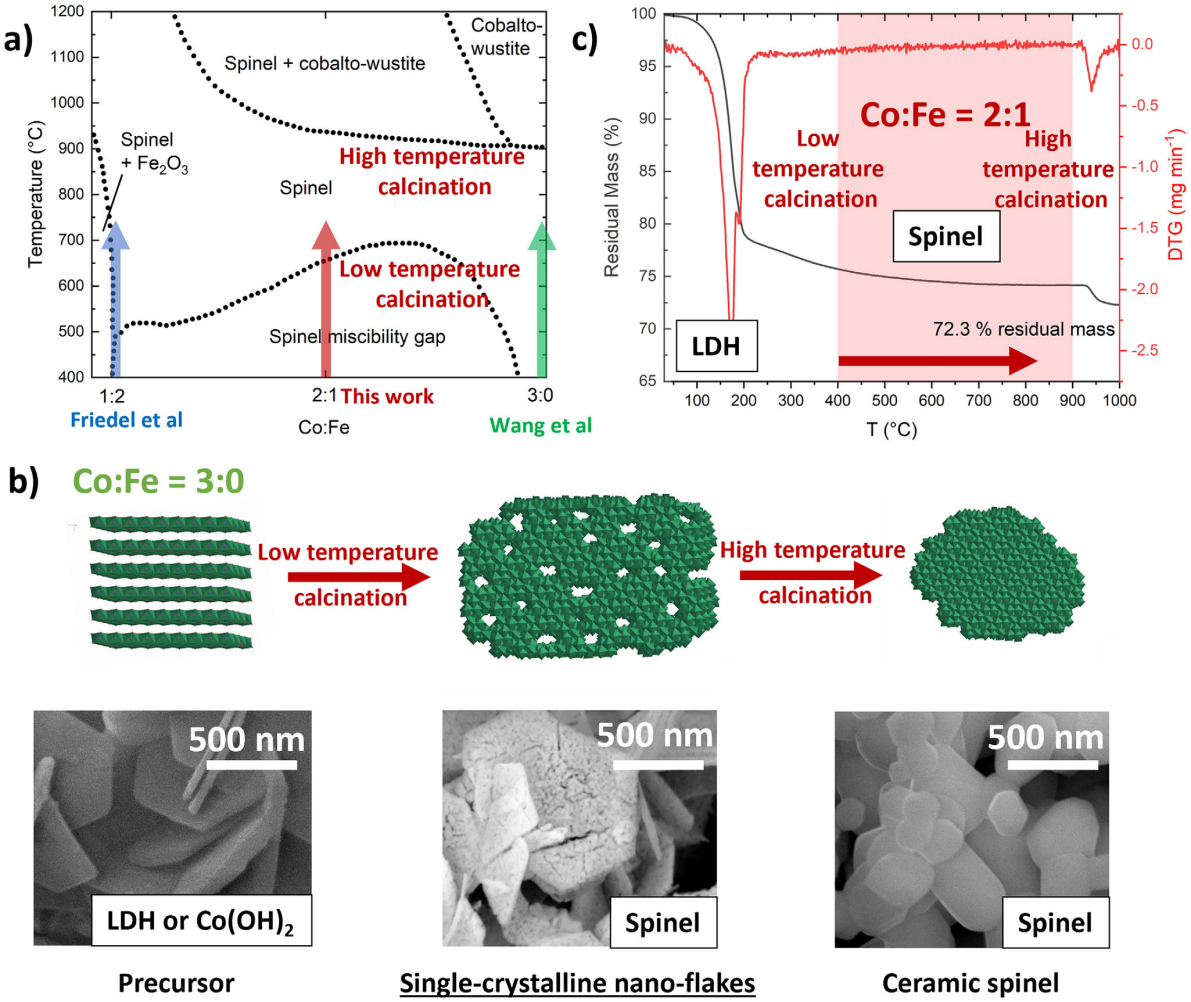
a) Schematic representation of the Fe─Co─O phase diagram, adapted from Zhang et al.,^[^ ^20^ ^]^ The positions of CoFe_2_O_4_, Co_2_FeO_4_, and Co_3_O_4_ in the phase diagram were added.^[^ ^21^, ^22^ ^]^ b) The crystalline precursor decomposition approach leading to single‐crystalline nano‐flakes and c) thermogravimetric analysis for identifying feasible temperature ranges for spinel formation.

The spinel structure is generally expressed as (A^II^)*_t_* (B^III^ *_2_*)*_o_* O_4_, where the divalent A cations occupy one eighth of the tetrahedral sites (*t)* and the trivalent B cations occupy half of the octahedral sites (*o)* in a face‐centered cubic structure of oxygen atoms.^[^ ^8^ ^]^ This structure is denoted as the normal spinel type. Inverse spinels are described by the formula (B^III^)*_t_* (A^II^,B^III^) *_o_* O_4_, in which the divalent A cations are in octahedral sites and the trivalent B cations are evenly distributed in tetrahedral and octahedral sites. Which spinel structure type is formed depends on the size of the cations, their ligand field stabilization and/or Madelung energy.^[^ ^9^ ^]^ Often an intermediate state is observed, which can be described as partially inverse by (A^II^_1−x_B^III^_x_)*_t_* (A^II^_x_B^III^_2−x_)*_o_* O_4_ with a degree of inversion x.^[^ ^10^, ^11^ ^]^ A prototypical example is the antiferromagnetic below 40 K (AFM) Co_3_O_4_, which crystallizes in the normal spinel structure. The substitution of two Co cations by Fe leads to the inverse spinel CoFe_2_O_4_, which is ferrimagnetic (FiM). The compound Co_2_FeO_4_, with a composition halfway between Co_3_O_4_ and CoFe_2_O_4_ is metastable at temperatures below 600 °C. In this regime the pseudo‐binary CoFe_2_O_4_ – Co_3_O_4_ phase diagram shows a large miscibility gap (Figure 1).^[^ ^12^, ^13^ ^]^ This miscibility gap causes the segregation of Co_2_FeO_4_ into an iron‐rich, mostly inverse, FiM CoFe_2_O_4_‐like phase and a cobalt‐rich, mostly normal, AFM Co_3_O_4_‐like phase by spinodal decomposition.^[^ ^14^ ^]^ A segregation process in Co_2_FeO_4_ nanoparticles was also observed by Xiang et al. during electrocatalysis.^[^ ^15^ ^]^ The position of Co_2_FeO_4_ inside the miscibility gap can also be used for engineering FiM‐AFM interfaces. Such FiM‐AFM interfaces can give rise to an interesting magnetic phenomenon, the exchange bias effect, which was first observed by Meiklejohn and Bean in core‐shell system consisting of a metallic cobalt core and an oxidized CoO shell.^[^ ^16^ ^]^ The exchange bias shifts the center of the hysteresis loop of a ferro‐ or ferrimagnet along the axis of the magnetic field. This effect is a consequence of the exchange coupling at the interface between the ferrimagnetic or ferromagnetic and antiferromagnetic domains.^[^ ^17^ ^]^ Moreover, it is essential in technologies such as magnetic sensors or magnetic‐storage applications.^[^ ^18^, ^19^ ^]^

Naturally, the interface between the FiM and AFM components is pivotal for exchange bias to occur, so increasing the interfacial area between AFM and FiM components in a material is desirable. Usually, two crystallographically distinct phases are neighboring each other, with one phase exhibiting AFM and the other exhibiting FiM properties, modifying the magnetic exchange interactions across the shared interface. Typical examples are core shell particles or multilayer thin films.^[^ ^23^, ^24^, ^25^, ^26^ ^]^ Also reported were approaches in which exchange bias was induced by interaction between two magnetic states in a single phase.^[^ ^27^, ^28^, ^29^ ^]^ On the other hand, it should be possible to increase the FiM‐AFM interface area at the nanoscale, while using the AFM and FiM phases crystallizing in the same general structure type, the spinel lattice. Such atypical exchange bias between two isostructural phases has to the best of our knowledge so far mainly been observed in natural minerals.^[^ ^30^, ^31^ ^]^ Even though related effects were observed by Rivas–Murias et al. in chemically synthesized cobalt ferrite nanocrystals, where exchange bias was induced by creating different metal to oxygen ratios, the latter was kept constant for each of our samples.^[^ ^32^ ^]^

We herein present a synthetic strategy to enhance the specific FiM‐AFM contact within crystalline spinel oxide flakes at the nanoscale and demonstrate its relevance for exchange bias. We employed a low‐temperature approach based on the recently described topotactic decomposition of a layered double hydroxide (LDH) precursor to obtain a nano‐sized and anisotropic platelet‐shaped spinel with a Co:Fe ratio of 2:1.^[^ ^33^ ^]^ As the LDH precursor does not show any miscibility issue in this composition range, compositions within the miscibility gap of the resulting spinel are readily accessible.^[^ ^33^ ^]^ The easily scalable thermal decomposition of the LDH precursor at only 400 °C yielded porous crystalline and metastable spinel nanoflakes, in which alternating FiM‐ and AFM mosaic domains are separated by epitactic solid–solid interfaces. As diffusion of atoms has been kinetically trapped at such low temperature, nanoscale domains form from the uniform precursor. This way, we are able to engineer the exchange bias effect usually only observed in thin films or core shell nanoparticles into a simple powder material, foregoing the requirement of complex synthesis or deposition procedures. The self‐assembly of the magnetic domains within one individual crystal and the thermal evolution of this unique microstructure was investigated by X‐ray diffraction (XRD), pair distribution functional (PDF) analysis, scanning electron microscopy (SEM), high resolution (scanning) transmission electron microscopy (HR‐(S)TEM), combined STEM‐energy dispersive X‐ray spectroscopy (STEM‐EDX), X‐ray absorption spectroscopy (XAS), Mössbauer spectroscopy, and magnetometry. To gain insight into the underlying properties, density functional theory calculations are performed with an on‐site Hubbard term (DFT + *U*).

## Results and Discussion

Metastable Co:Fe = 2:1 spinel‐type oxides have been prepared by the thermal decomposition of crystalline precursors as described previously for Co:Fe = 1:2, yielding CoFe_2_O_4_ (from a (Co^II^_0.33_Fe^II^_0.33_Fe^III^_0.33_)(OH)_2_(CO_3_)_0.17_ × *m*H_2_O LDH‐precursor, blue arrow in Figure 1a) and Co:Fe = 3:0 yielding Co_3_O_4_, (from a Co^II^_2_(OH)_2_ hydroxide precursor, green arrow in Figure 1a).^[^ ^33^ ^]^ For this work, a phase‐pure LDH precursor of the general sum formula (Co^II^_0.67_Fe^III^_0.33_)(OH)_2_(CO_3_)_0.17_ × *m*H_2_O (Figure S1a) has also been synthesized with a composition falling into the miscibility gap of the spinel phase diagram (red arrow Figure 1a). The precursor exhibits the typical intergrown platelet morphology of co‐precipitated LDH materials with a platelet size of around 0.5 µm (Figure S1b).^[^ ^33^ ^]^

Next, this LDH precursor has been thermally decomposed in air to yield the desired Co_2_FeO_4_ spinel. Upon calcination in the presence of oxygen, half of the Co^2+^ cations are oxidized to Co^3+^ to adjust the trivalent to bivalent cation ratio needed for the formation of a spinel without the need for extended cation diffusion. Indeed, Co K‐edge X‐ray absorption near‐edge structure (XANES) measurements indicated an increase in the average Co oxidation state upon calcination, although a slightly higher value than the expected 2.5 was found (Figures S18 and S19). Investigation of the metal L_3_ edges furthermore showed the presence of Co^2+^ and ruled out the presence of Fe^2+^ in the near‐surface region (Figures S19–S21). A detailed discussion of the XAS data can be found in the Supporting Information. Figure 1c shows the thermogravimetric (TG) analysis of the precursor in synthetic air from which the complete precursor decomposition and the thermal re‐reduction of the formed trivalent cobalt at high temperature can be derived. The LDH decomposition starts at temperatures around 200 °C, which is in line with literature.^[^ ^34^ ^]^ After calcination at 400 °C the mass loss remains stable up to 922 °C, where the thermal reduction of Co^3+^ cations in the spinel and the conversion into cobalto‐wustite takes place, which is in good agreement with the phase diagram (Figure 1a). Therefore, temperatures from 400 to 900 °C were selected as suitable calcination temperatures in order to comparatively assess the stability and the magnetic properties of the synthesized spinel products.

**Figure 2:**
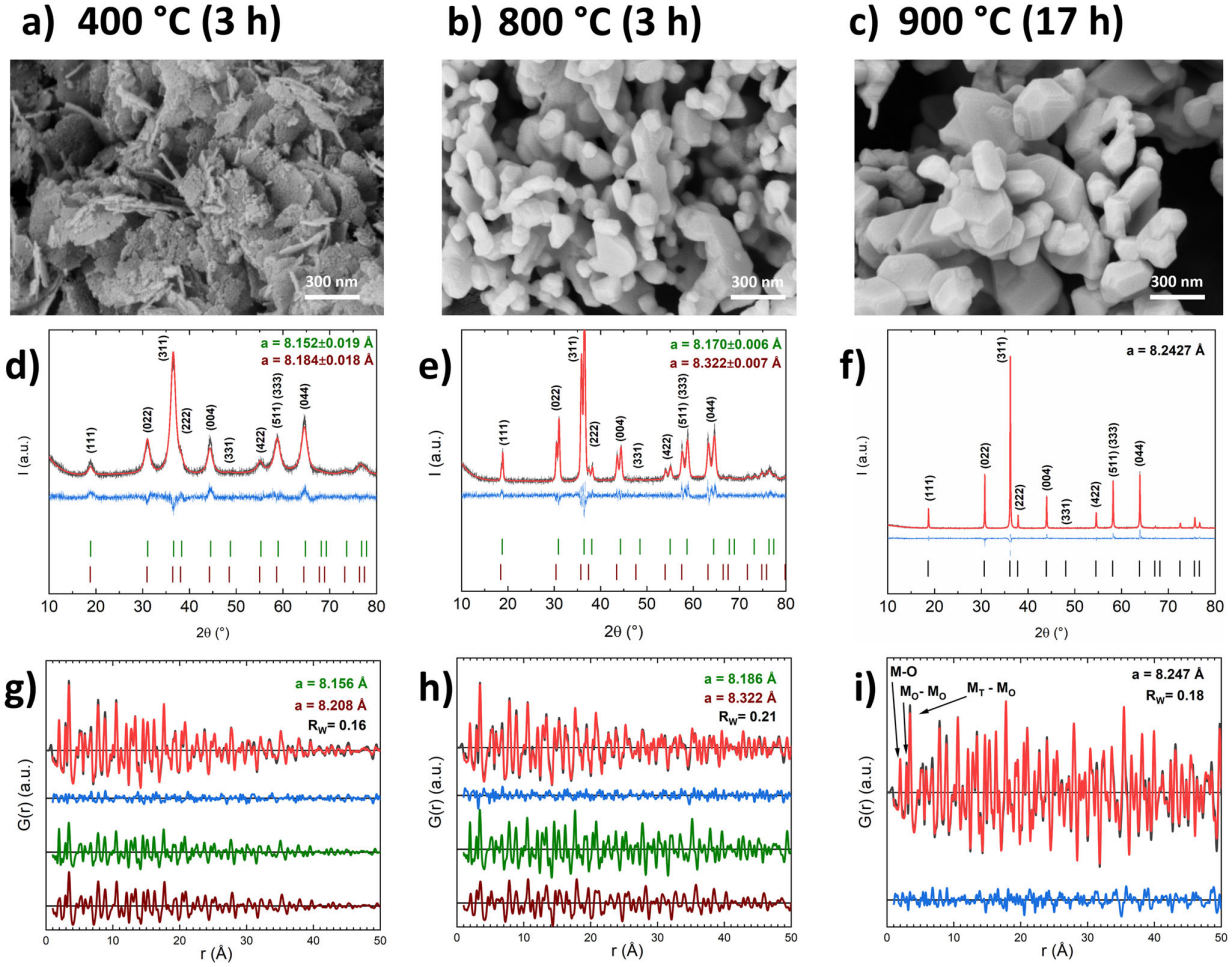
SEM micrographs a)–c), X‐ray diffraction patterns d)–f) and PDF analysis g)–i) of the samples after thermal treatment at three different calcination temperatures: 400 °C (left), 800 °C (center), 900 °C (right). PXRD refinements (red curve) with overlapping experimental data (black curve) are shown together with the difference curve (blue, in offset) and are additionally indexed with Bragg reflections of the cobalt‐rich phase (green) and iron‐rich phase (brown) for 400 and 800 °C samples, and with Co_2_FeO_4_ for 900 °C. PDF refinements are shown with experimental PDF (black) and fit (red), as well as in offset the difference (blue), cobalt‐rich phase (green) and iron‐rich phase (brown).

Thermal decomposition of the LDH precursor was conducted in this temperature range in 50 °C steps for 3 h, while samples calcined at 400, 800, and 900 °C have been selected as representatives of samples in the miscibility gap (400 °C), at the border of the miscibility gap (800 °C), and in the stable single‐phase regime (900 °C). The SEM images of these three calcination products are shown in Figure 2a–c. After calcination at 400 °C, porous pseudomorphs of the LDH precursor platelets are obtained, as expected from the topotactic decomposition (Figure 2a). After calcination at 800 °C, sintering causes the depletion of pores and loss of the anisotropic morphology (Figure 2b). This effect is even more pronounced after calcination at 900 °C (Figure 2c). For the latter sample, a longer dwell time of 17 h was needed for a complete “ceramic” homogenization yielding a phase pure Co_2_FeO_4_ sample after passing through the miscibility gap as determined by XRD (Figure 2f).^[^ ^14^ ^]^

Rietveld refined powder XRD patterns (Figure 2d–f) and PDF analysis (Figure 2g–i) of the calcination series show that the samples calcined at 800 and 900 °C are highly crystalline (Figure 2e,f). A phase mixture is observed for the sample calcined at 800 °C (Figure 2e) despite the fact that the calcination temperature was above the miscibility gap indicated in the phase diagram.^[^ ^6^ ^]^ This shows that the sample has not reached thermodynamic equilibrium. The cubic lattice parameters *a* derived from Rietveld refinement agrees with the phase separation into an iron‐rich spinel (*a *= 8.3208 Å) and a cobalt‐rich spinel (*a *= 8.1683 Å). A single Co_2_FeO_4_ phase was obtained only after prolonged annealing at 900 °C (Figure 2f). After calcination at 900 °C for 17 h no splitting in the reflections can be observed and the refined lattice parameter (*a *= 8.2427 Å) agrees with that for phase pure Co_2_FeO_4_ reported in the literature, indicating that the thermodynamically stable state has been reached.^[^ ^14^ ^]^ Fe K‐edge XANES analysis revealed that Fe^3+^ occupies the octahedral sites of the spinel structure, the average oxidation state of Co was 2.6 ± 0.1 and that of Fe 3.1 ± 0.2. Within the margins of error, these values were also confirmed for the samples calcined at lower temperatures (see SI for further discussion). To further investigate the short‐ and intermediate‐range order of the samples in greater detail, PDF measurements were performed. For the sample calcined at 800 °C (Figure 2h), a biphasic model with two spinels led to a satisfying fit, and for the 900 °C sample a one‐spinel model was used for satisfying results (Figure 2i), corroborating the findings from Rietveld refined XRD data. In addition, the PDF analysis indicates a similar trend in the lattice parameter as provided by the XRD analysis (Table 1). The 400 °C sample shows lower crystallinity in the PXRD data than the 800 °C sample. Rietveld refinement indicated two segregated spinel phases with significantly different lattice parameters. Interestingly, the lattice parameter for the iron‐enriched phase is significantly smaller than expected, which will be discussed below. In the PDF, a biphasic model resulted in an improved fit compared to one phase only, while the crystallite sizes were refined to about 7 nm for both phases. For the 800 °C sample these domain sizes have increased to 13 and 14 nm for the iron‐rich and cobalt‐rich phase, respectively. Further sample characterization was carried out via Raman spectroscopy (Figures S15–S17), agreeing with estimations of phase compositions discussed in detail in the Supporting Information, based on PXRD, magnetic transition temperatures, and EDX.

**Table 1:** Lattice parameter and crystallite sizes derived from XRD, PDF, and HR‐TEM. The error for the lattice parameter from XRD were taken from Rietveld refinement (performed with the TOPAS software). Error for the lattice parameters from TEM result from the confidence interval of the fit. Domain size was determined as the volume‐weight mean column height from integral breath.

*T*_calc_	Phase	Lattice parameter XRD (Å)	Lattice parameter PDF (Å)	Lattice parameter HR‐TEM (Å)	Crystallite size Lvol‐IB (nm)	Crystallite size PDF (nm)
400 °C	Co‐rich	8.152 ± 0.019	8.156	8.07 ± 0.02	5	7
	Fe‐rich	8.184 ± 0.018	8.208	8.13 ± 0.02	4	7
800 °C	Co‐rich	8.170 ± 0.006	8.186		16	14
	Fe‐rich	8.322 ± 0.007	8.322		18	13
900 °C	Co_2_FeO_4_	8.2427 ± 0.0007	8.247		bulk‐like (>50)	bulk‐like

**Figure 3:**
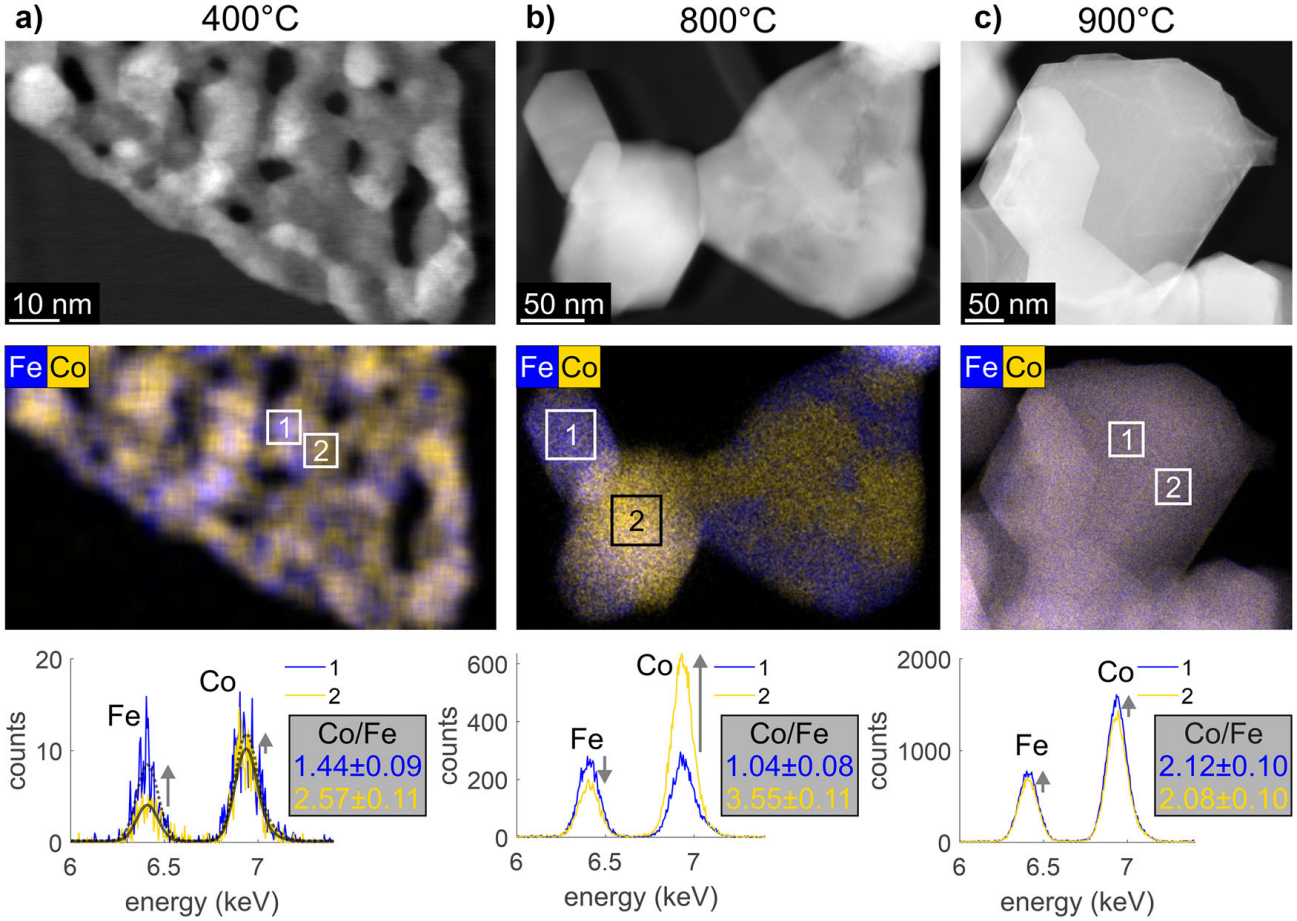
STEM images of all three samples (upper row), EDX maps with Fe represented in blue and cobalt represented in yellow (middle row) and the cobalt to iron atomic ratio derived from EDX spectra extracted from the two different areas 1 and 2 (lower row).

To unravel the spatial distribution of the different phases and the composition of this novel material, STEM‐EDX measurements were conducted. Figure 3 shows STEM images and the corresponding EDX maps with spectra obtained for the indicated regions of the images (rectangles labelled by 1 and 2). For the sample calcined at 800 °C, the segregation into cobalt‐rich (yellow) and iron‐rich (blue) domains at sizes of several tens of nanometers is clearly evident and in agreement with a state “in the miscibility gap” already confirmed by XRD and PDF results. After prolonged thermal treatment at 900 °C, the phase segregation has vanished and the nominal atomic ratio of 2 to 1 is uniformly obtained (see inset in Figure 3c). For the 400 °C spinel however, a multitude of nano domains of sizes around only 5 nm are observed in the EDX map showing the lateral distribution of Co and Fe within the nano‐flake (Figure 3a). This dimension is well in line with the PDF domain sizes of about 7 nm. These domains are either enriched in cobalt or in iron, as shown in the middle and lower row of Figure 3. Thus, the STEM‐EDX results confirm the co‐existence of two different phases as concluded from the XRD and PDF data. According to the difference in lattice parameters and as evident from the EDX map, these two phases contain different amounts of Co and Fe and share interfaces with each other. However, due to the perfect intermixing of the cations in the precursor and the limited diffusion at the relatively low calcination temperature, the spinel is segregated only on the level of very small nano‐domains.

**Figure 4:**
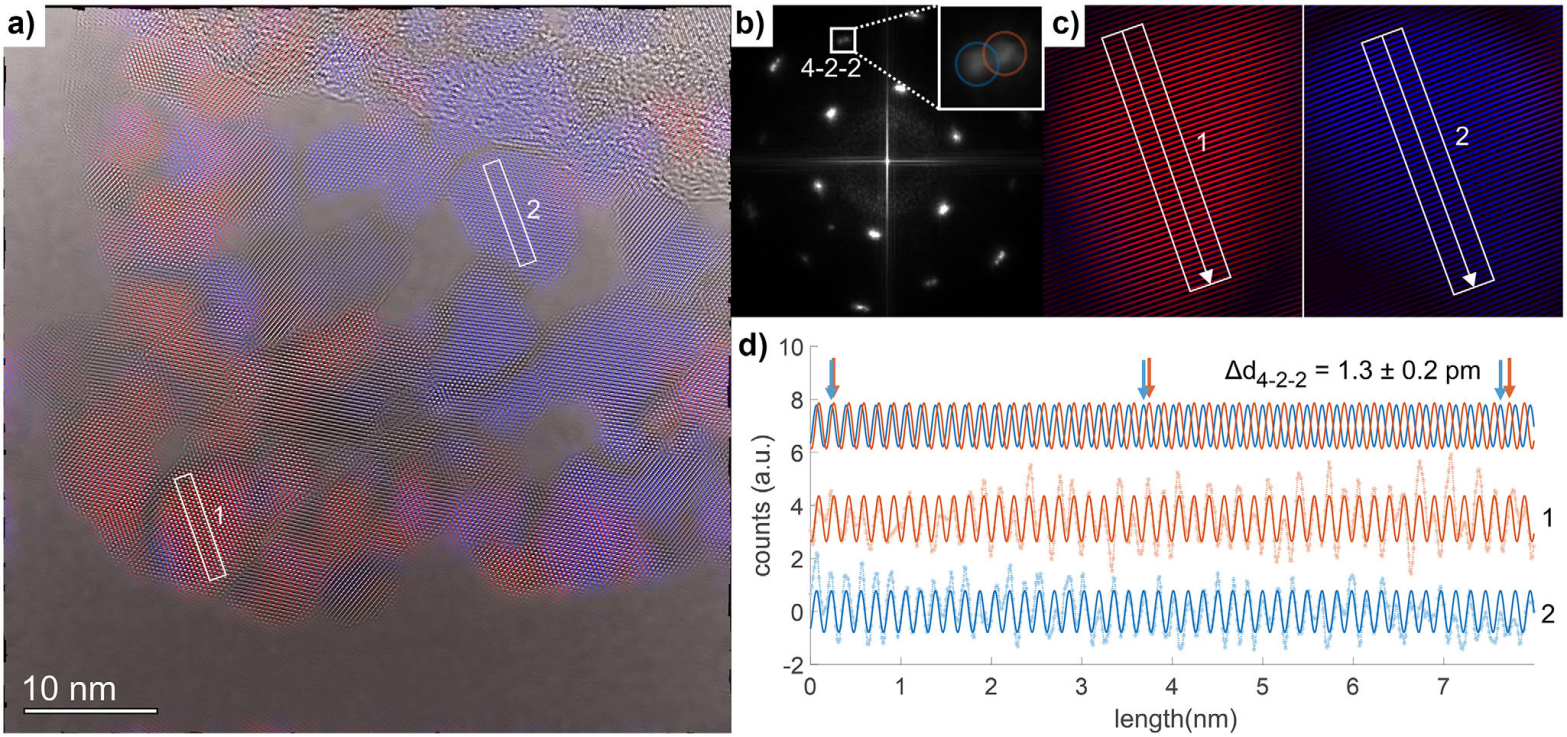
HR‐TEM images with superimposed iFFT image of the 400 °C sample a), FFT pattern b), zoomed in areas 1 and 2 c), and determination of the lattice parameter in real space d), extracted from line profiles from region 1 and 2, as indicated by the white arrows in (c).

As mentioned above, the transformation from LDH precursors to spinels under mild conditions usually occurs topotactically, leading to single crystalline porous spinel platelets with their [111] direction oriented parallel to the stacking *c*‐axis of the LDH (see Figure 1).^[^ ^21^ ^]^ To study the crystallographic ordering and orientation in the 400 °C sample, consisting of two spinel phases with different cobalt to iron ratios, HR‐TEM imaging on single platelets such as shown in Figure 4 was performed. The HR‐TEM micrograph in Figure 4 shows atomic resolution and indicates a high and unperturbed crystalline ordering in the whole nano‐flake, which is interrupted, but not changed in orientation by the pores (see also Figure S2). Consequently, even though there are two phases present, the Fast Fourier Transform (FFT) of the HR‐TEM image in Figure 4b resembles that of a single‐crystalline platelet as previously observed for the phase‐pure spinels Co_3_O_4_ and CoFe_2_O_4_ synthesized by this approach.^[^ ^21^, ^33^ ^]^ This is remarkable, as it implies that all nano‐domains share the same crystallographic orientation (with [111] being the zone axis direction) and that a multitude of epitactic phase boundaries is present within each platelet, due to the very small size of the nano domains. Nevertheless, due to the different compositions of the nano domains, a description as a single crystal would not be correct. This is underlined by the detailed investigation of the FFT pattern, which showed a small splitting of the 4$\bar{22}$ reflections as a result of the difference in spinel lattice parameter in the two domain types (highlighted by the red and blue circle in the inset of Figure 4b). Therefore, we propose a “single crystalline like” nature, with an intermediate state between single crystal and mesocrystal, as the latter is defined as an arrangement from individual nanoparticles and usually exhibits spatial separation between the domains,^[^ ^35^ ^]^ which is not the case here. Inverse Fast Fourier transformation (iFFT) of the two contributions of the 4$\bar{22}$ reflection unraveled the local distribution of the different domains. The iFFT images are superimposed with the HR‐TEM image as red and blue components in Figure 4a. Most of these were found well‐separated from each other, having comparable domain sizes as found for the iron‐ and cobalt‐rich areas by EDX mapping. Two representative domains were selected to determine the interatomic distances in real space (Figure 4d), from which the lattice parameters for both domains were calculated from the HR‐TEM image (8.0686 and 8.1323 Å) and summarized in Table 1.

Table 1 summarizes and compares the lattice parameters, and crystallite sizes derived from XRD, PDF, and HR‐TEM analysis. Even though all methods result in slightly different absolute values, the overall trend for all samples is the same. For the sample obtained at 400 °C, with its peculiar microstructure, the relative difference found between the lower and higher value is very similar independent of the method used (XRD: 0.4%, PDF: 0.6%, and TEM: 0.8%). Given this consistency, the sample obtained at 400 °C can be described as holey single‐crystalline‐like nano‐flakes with unique structural properties between those of a single and mesocrystal.

**Figure 5:**
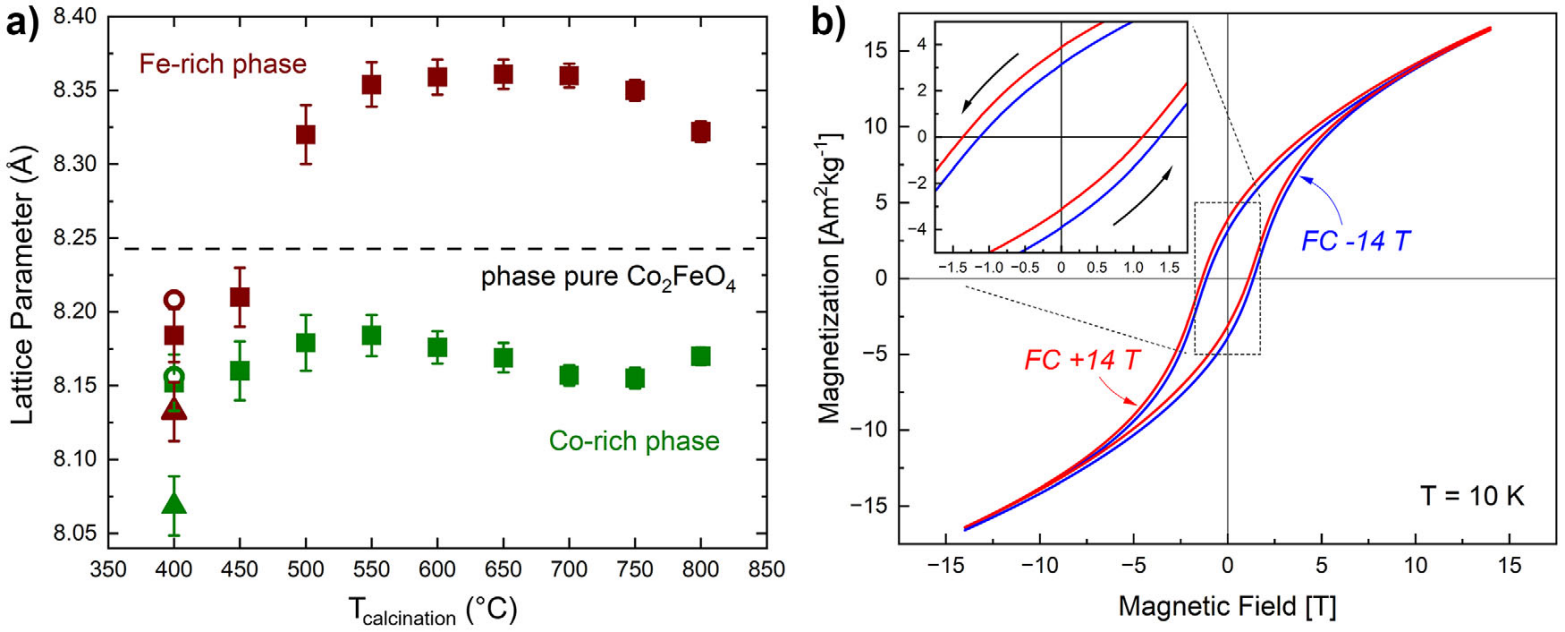
a) Lattice parameter of the cobalt (green)‐ and iron (brown)‐ rich phases for the whole calcination series were determined by Rietveld refinement and are represented by squares. The lattice parameter for the 400 °C sample was also obtained from PDF data (open circles) and HR‐TEM (triangles). b) Magnetic hysteresis loops of the sample calcined at 400 °C, measured subsequent to field cooling (FC) from 390 K at 14 T (red) and ‐14 T (blue).

Interestingly, all analytical techniques indicate that the lattice parameter of the iron‐rich phase for the sample calcined at 400 °C is significantly smaller, compared to expectations from the spinodal decomposition.^[^ ^6^ ^]^ Figure 5a shows the evolution of the lattice parameter found by Rietveld refinement with calcination temperature. The weighted average of the observed lattice parameters of the cobalt‐rich and the iron‐rich phases should result in the literature value for Co_2_FeO_4_^[^ ^14^ ^]^ (dotted black line in Figure 5a), here being represented by the value for the sample calcined at 900 °C. This is only the case for calcination temperatures above 550 °C. Below, the lattice parameter of the Fe‐rich phase is surprisingly small, which we attribute to the unique above‐described meta‐stable state of the samples. Variations in bulk composition between the samples, an incomplete LDH transformation or another side phase being present could be ruled out as an explanation as discussed in detail in the SI.

**Figure 6:**
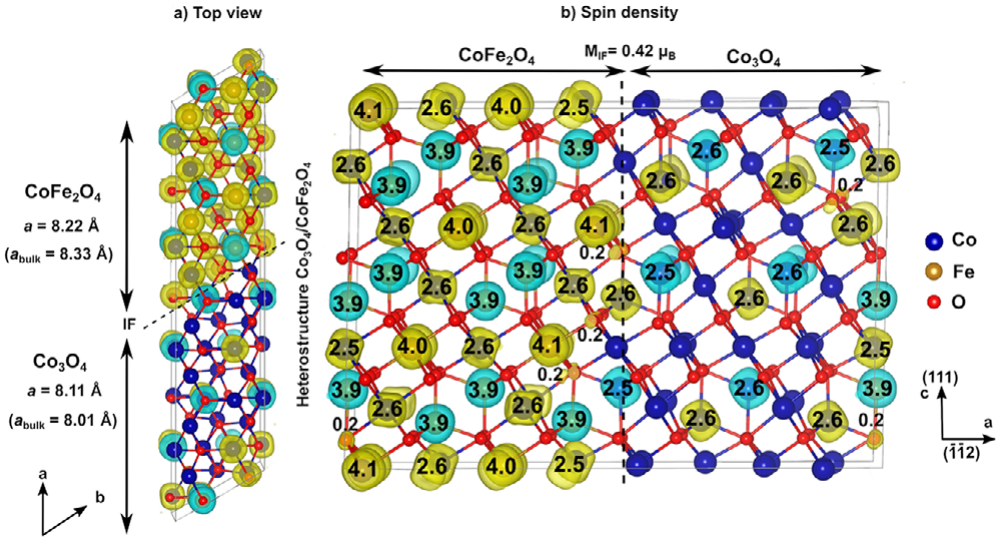
a) Top and b) side view of the structure and spin density of (111)‐oriented heterostructure of CoFe_2_O_4_ and Co_3_O_4_ with an interface along the hexagonal *a* direction. Yellow and cyan colors represent the majority and minority spin density, respectively. *M*_IF_ presents the total magnetic moment of the interface layer in the heterostructure in μ_B_. *a* and *a*_bulk_ denote the lattice parameter of the CoFe_2_O_4_ and Co_3_O_4_ part of the heterostructure and the related bulk lattice constants, respectively.

To investigate the origin of the decreased lattice parameter of the iron‐rich phase in the 400 °C sample and to gain deeper insight in the experimentally observed phase separation in Co‐ and Fe‐ rich regions, density functional theory (DFT) calculations were performed with the VASP code and the PBEsol exchange correlation functional^[^ ^36^ ^]^ within the generalized‐gradient approximation and an additional on‐site Hubbard *U* = 3 eV term on both Co and Fe 3*d* states. For the calculations, we considered the bulk phases of Co_2_FeO_4_, the end members Co_3_O_4_ and CoFe_2_O_4_, as well as interfaces between the end members Co_3_O_4_ and CoFe_2_O_4_ with different crystallographic orientations. To model as close as possible the (111)‐oriented platelets with Co‐ and Fe‐rich regions, as observed in the HR‐TEM images, we have considered a heterostructure of (111)‐oriented CoFe_2_O_4_ and Co_3_O_4_ with a lateral interface along the hexagonal *a*‐direction [$\bar{11}$2]. Other heterostructures with interfaces along the [111]‐direction or oriented along [001] are discussed in the SI. The structural optimization of the Co_3_O_4_/CoFe_2_O_4_ heterostructure resulted in a decreased volume of 556.63 Å^3^ in the Fe‐rich part (CoFe_2_O_4_), which corresponds to a cubic lattice constant of 8.22 Å compared to the bulk volume of 577.13 Å^3^ (*a* = 8.33 Å). The trend of the calculated values is similar to the experimental values found for the sample calcined at 400 °C (Figure 6). The Co‐rich part of the heterostructure, Co_3_O_4_, has a volume of 533.033 Å^3^ (*a *= 8.11 Å) in agreement with the measurements (Figure 6), which is enhanced compared to the Co_3_O_4_ bulk volume of 513.026 Å^3^ (*a* = 8.01 Å). For comparison, the calculated lattice constant of bulk Co_2_FeO_4_ is 8.19 Å. Furthermore, the Co_3_O_4_/CoFe_2_O_4_(111) heterostructure is found to be energetically more stable by −0.15 eV/f.u. than the nominal stoichiometry Co_2_FeO_4_, which indicates that the spinodal decomposition into two phases is favored. The decreased lattice parameter of CoFe_2_O_4_ can be explained by the substantially smaller bulk modulus of CoFe_2_O_4_ (187.7 GPa) compared to Co_3_O_4_ (232.9 GPa) and Co_2_FeO_4_ (238 GPa), indicating a stronger compressibility of the CoFe_2_O_4_ spinel, which represents the iron‐rich domains, in the Co_3_O_4_‐CoFe_2_O_4_ spinodal decomposition. The compression of the Fe‐richer domains is realized due to the presence of many small alternating nano‐domains, separated by epitactic interfaces. This unique state is thermally destroyed at higher calcination temperatures allowing enhanced atom mobility, i.e., around 450 to 500 °C in this case.

In addition to these structural consequences, the unique morphology in the low‐calcined samples is in particular interesting as the local compositional variations give rise to a multitude of interfaces between two phases with distinct magnetic properties. To further investigate the correlation of structural and magnetic properties, Mössbauer spectroscopy and magnetometry as well as DFT + *U* calculations of the magnetic properties in the bulk and at the interface were performed. The side view of the Co_3_O_4_/CoFe_2_O_4_(111) heterostructures together with the spin density and magnetic moments obtained from DFT + *U*, shown in Figure 6a,b, indicates subtle changes of the magnetic moments in the vicinity of the interface: a slight enhancement of the magnetic moment of Fe^3+^ at the octahedral sites to 4.1 μ_B_ (bulk value: 4.0 μ_B_) and a slight reduction of the magnetic moment of Co^2+^ at the tetrahedral sites to 2.5 μ_B_ close to the interface, the remaining Co^2+^ ions having a bulk‐like magnetic moment of 2.6 μ_B_. Additionally, we observe a noticeable spin‐polarization of 0.2 μ_B_ at oxygen sites close to the interface. The layer‐ and element‐resolved density of states, presented in Figure S4d gives further insight into the band alignment and electronic reconstruction at the interface. A noticeable exchange splitting in the majority and minority channel is obtained in the interface Co_3_O_4_ layer resulting in 0.42 μ_B_ net magnetization. Similarly, the magnetization is also enhanced in the interface region of the CoFe_2_O_4_ part.

**Figure 7:**
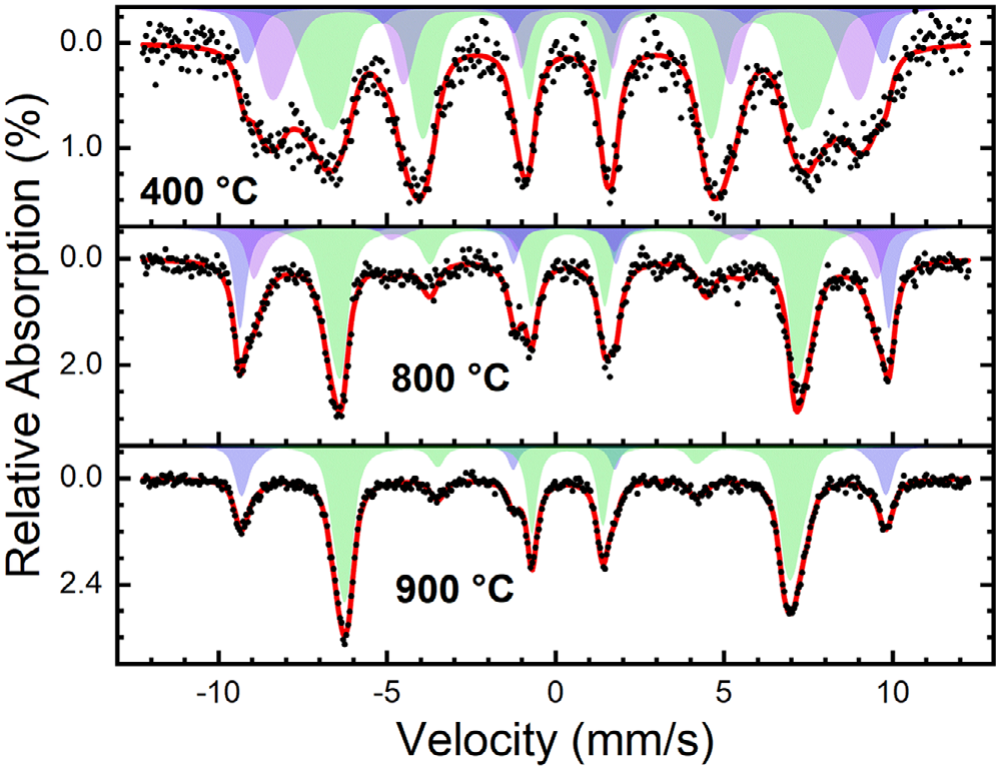
Mössbauer spectra recorded at 4.3 K and an applied field of 10 T parallel to the γ‐ray propagation direction on the representative samples calcinated at 400, 800, and 900 °C. Experimental data (black dots) is shown together with a theoretical data fit (red line) composed of several superimposed subspectra, representing Fe atoms residing on B‐ (green) and A‐sites of the spinel lattice, with latter being assigned to contributions from Fe^3+^ ions in Fe‐rich (blue) and Co‐rich (violet) environments.

Mössbauer spectroscopy was performed to analyze the average cation distribution on different crystallographic positions, showing distinct variations for different annealing temperatures. Figure 7 shows the Mössbauer spectra for the three representative samples calcined at 400, 800, and 900 °C. Overall, Mössbauer spectroscopy agrees very well with all other characterization methods. The presence of Fe^3+^ in iron‐rich and cobalt‐rich surroundings is indicated by the appearance of a third subspectrum, when comparing the single‐phase material (900 °C) to the one obtained at mild calcination temperatures. At the same time, one observes a marked increase in magnetic frustration at low calcination temperatures, which could be assigned to a pinning of the magnetic moments of the FiM iron‐rich regions, due to coupling to adjacent AFM Co_3_O_4_‐like domains. In agreement with results from the bulk‐sensitive Mössbauer spectroscopy method, XAS data indicated the presence of Fe^3+^ only. A more detailed discussion and spectra for all calcination temperatures can be found in the Supporting Information (Figure S10).

For the 400 °C sample, extensive field‐ and temperature‐dependent magnetization measurements were performed. Results of the general magnetic characterization are illustrated in greater detail in the Figures S11–S13. All analytical techniques indicate the importance of interfacial effects between Co‐ and Fe‐rich regions and support their AFM and FiM behavior accordingly. Thus, field cooling experiments were conducted to investigate possible coupling effects. To make sure to pass the Néel temperature of the cobalt‐rich phase during cooling, which was characterized in ZFC‐FC magnetization experiments (Figure S13), the sample was heated to 390 K and subsequently cooled to 10 K at 14 T and ‐14 T respectively before recording M(H) curves (see Figure 5b).^[^ ^13^ ^]^ The exact measurement protocol can be found in the methods section in the Supporting Information. The recorded M(H) curve exhibits a shift of 115 mT, which represents an exchange bias effect with the temperature dependence of the exchange bias field shown in more detail in Figure S14. Such an effect is usually only found within thin films, core‐shell nanoparticles or natural minerals, and has not yet been observed in powders composed of particles with the aforementioned platelet morphology, synthesized using the described simple wet‐chemical method followed by mild calcination. The increase of magnetization at the interface between the nanodomains predicted by theory is assumed to be the reason for this phenomenon (see Figure 6 and further discussion in the Supporting Information). The observed exchange bias can therefore be assigned to the metastable state of the strongly intermixed AFM and FiM regions and possibly to the unusual compression of the crystal structure of the Fe‐rich phase showing enhanced interface magnetization.

## Conclusion

Within this work, a sample series was synthesized by applying the crystalline precursor decomposition approach to a LDH precursor with a Co:Fe ratio of 2:1 at temperatures in the range of 400 to 900 °C. The Co:Fe ratio lies within the miscibility gap of the spinel phase diagram, leading to samples with a differing degree of phase segregation. This approach led to the formation of a unique sample morphology at the mildest calcination temperature of 400 °C. Porous single‐crystalline‐like nano‐flakes with spatial compositional fluctuations on the nanoscale (ca. 5 nm) were obtained and thoroughly examined by state‐of‐the‐art characterization methods. XRD, PDF, and HR‐TEM analysis revealed a contraction of the lattice parameter of the Fe‐rich phase as a result of this peculiar metastable microstructure. The latter can be explained by the DFT + *U* results with the higher compressibility of the Fe‐rich phase at the interfaces, exhibiting enhanced magnetization. In addition, investigation of the magnetic properties showed an exchange bias effect, which we ascribe to strong coupling of adjacent AFM cobalt‐rich and FiM iron‐rich regions within the platelets, as also indicated by Mössbauer spectroscopy data. Such magnetic properties form the basis for different applications and are usually only observed in materials with designed interfaces, not self‐assembled in powders. We believe that this facile synthetic approach can be transferred to other oxides and holds potential for unlocking new magnetic properties via self‐assembled nanostructures.

## Supporting Information

The authors have cited additional references within the Supporting Information.

## Conflict of Interests

The authors declare no conflict of interest.

## Supporting information



Supporting Information

## Data Availability

The data that support the findings of this study are available from the corresponding author upon reasonable request.

## References

[1] E. Budiyanto , M. Yu , M. Chen , S. DeBeer , O. Rüdiger , H. Tüysüz , ACS Appl Energ Mater 2020, 3, 8583–8594.

[2] S. Y. Srinivasan , K. M. Paknikar , D. Bodas , V. Gajbhiye , Nanomedicine 2018, 13, 1221–1238. 29882719 10.2217/nnm-2017-0379

[3] Q. Song , Z. J. Zhang , J. Am. Chem. Soc. 2004, 126, 6164–6168. 15137781 10.1021/ja049931r

[4] S. Jauhar , J. Kaur , A. Goyal , S. Singhal , RSC Adv. 2016, 6, 97694–97719.

[5] N. Debnath , T. Kawaguchi , W. Kumasaka , H. Das , K. Shinozaki , N. Sakamoto , H. Suzuki , N. Wakiya , J. Magn. Magn. Mater. 2017, 432, 391–395. 10.1080/14686996.2018.1482520 PMC6041787 30013695

[6] T. M. C. Dinh , A. Barnabe , M. A. Bui , C. Josse , T. Hungria , C. Bonningue , L. Presmanes , P. Tailhades , Cryst. Eng. Comm. 2018, 20, 6146–6155.

[7] O. Boytsova , O. Makarevich , D. Sharovarov , A. Makarevich , Inorg. Mater. 2022, 58, 673–686.

[8] O. Vozniuk , T. Tabanelli , N. Tanchoux , J. M. M. Millet , S. Albonetti , F. Di Renzo , F. Cavani , Catalysts 2018, 8, 332.

[9] A. Seko , K. Yuge , F. Oba , A. Kuwabara , I. Tanaka , Phys. Rev. B 2006, 73, 184117.

[10] Y. Hou , Y. Zhao , Z. Liu , H. Yu , X. Zhong , W. Qiu , D. Zeng , L. Wen , J. Phys. D: Appl. Phys. 2010, 43, 445003.

[11] C. Janiak , H.‐J. Meyer , D. Gudat , P. Kurz , in Riedel Moderne Anorganische Chemie, de Gruyter, Berlin/Boston 2018.

[12] I.‐H. Jung , S. A. Decterov , A. D. Pelton , H.‐M. Kim , Y.‐B. Kang , Acta Mater. 2004, 52, 507–519.

[13] M. Takahashi , M. E. Fine , J. Am. Ceram. Soc. 1970, 53, 633–634.

[14] T. A. S. Ferreira , J. C. Waerenborgh , M. H. R. M. Mendonca , M. R. Nunes , F. M. Costa , Solid State Sci. 2003, 5, 383–392.

[15] W. Xiang , N. Yang , X. Li , J. Linnemann , U. Hagemann , O. Ruediger , M. Heidelmann , T. Falk , M. Aramini , S. DeBeer , M. Muhler , K. Tschulik , T. Li , Nat. Commun. 2022, 13, 1–14. 35013310 10.1038/s41467-021-27788-2 PMC8748757

[16] W. H. Meiklejohn , C. P. Bean , Phys. Rev. 1956, 102, 1413–1414.

[17] K. M. Krishnan , Fundamentals and applications of magnetic materials, Oxford University Press, Oxford 2016.

[18] V. Skumryev , S. Stoyanov , Y. Zhang , G. Hadjipanayis , D. Givord , J. Nogués , Nature 2003, 423, 850–853. 12815426 10.1038/nature01687

[19] X. He , Y. Wang , N. Wu , A. N. Caruso , E. Vescovo , K. D. Belashchenko , P. A. Dowben , C. Binek , Nat. Mater. 2010, 9, 579–585. 20562879 10.1038/nmat2785

[20] W.‐W. Zhang , M. Chen , Calphad 2013, 41, 76–88.

[21] K. F. Ortega , S. Anke , S. Salamon , F. zcan , J. Heese , C. Andronescu , J. Landers , H. Wende , W. Schuhmann , M. Muhler , T. Lunkenbein , M. Behrens , Chem. ‐ Eur. J. 2017, 23, 12443–12449. 28657661 10.1002/chem.201702248

[22] J. Q. Wang , G. D. Du , R. Zeng , B. Niu , Z. X. Chen , Z. P. Guo , S. X. Dou , Electrochim. Acta 2010, 55, 4805–4811.

[23] M.‐H. Phan , J. Alonso , H. Khurshid , P. Lampen‐Kelley , S. Chandra , K. Stojak Repa , Z. Nemati , R. Das , Ó. Iglesias , H. Srikanth , Nanomaterials 2016, 6, 221. 28335349 10.3390/nano6110221 PMC5245749

[24] G. Salazar‐Alvarez , J. Sort , S. Surinach , M. D. Baró , J. Nogués , J. Am. Chem. Soc. 2007, 129, 9102–9108. 17595081 10.1021/ja0714282

[25] L. W. Martin , Y.‐H. Chu , M. B. Holcomb , M. Huijben , P. Yu , S.‐J. Han , D. Lee , S. X. Wang , R. Ramesh , Nano Lett. 2008, 8, 2050–2055. 18547121 10.1021/nl801391m

[26] Z.‐A. Li , N. Fontaíña‐Troitiño , A. Kovács , S. Liébana‐Viñas , M. Spasova , R. E. Dunin‐Borkowski , M. Müller , D. Doennig , R. Pentcheva , M. Farle , V. Salgueiriño , Sci. Rep. 2015, 5, 7997. 25613569 10.1038/srep07997 PMC4303864

[27] S. Middey , S. Jana , S. Ray , J. Appl. Phys. 2010, 108. 10.1088/0953-8984/22/34/346004 21403269

[28] M. Muroi , P. McCormick , R. Street , Rev. Adv. Mater. Sci 2003, 5, 76–81.

[29] X. Huang , J. Ding , G. Zhang , Y. Hou , Y. Yao , X. Li , Phys. Rev. B 2008, 78, 224408.

[30] S. A. McEnroe , B. Carter‐Stiglitz , R. J. Harrison , P. Robinson , K. Fabian , C. McCammon , Nat. Nanotechnol. 2007, 2, 631–634. 18654388 10.1038/nnano.2007.292

[31] R. Pentcheva , H. S. Nabi , Phys. Rev. B 2008, 77, 172405.

[32] B. Rivas‐Murias , M. Testa‐Anta , A. S. Skorikov , M. Comesaña‐Hermo , S. Bals , V. Salgueiriño , Nano Lett. 2023, 23, 1688–1695. 36848327 10.1021/acs.nanolett.2c04268 PMC10848284

[33] A. Rabe , J. Büker , S. Salamon , A. Koul , U. Hagemann , J. Landers , K. Friedel Ortega , B. Peng , M. Muhler , H. Wende , W. Schuhmann , M. Behrens , Chem. ‐ Eur. J. 2021, 27, 17038–17048. 34596277 10.1002/chem.202102400 PMC9298119

[34] L. Pesic , S. Salipurovic , V. Markovic , D. Vucelic , W. Kagunya , W. Jones , J. Mater. Chem. 1992, 2, 1069.

[35] E. V. Sturm , H. Cölfen , Chem. Soc. Rev. 2016, 45, 5821–5833. 27503260 10.1039/c6cs00208k

[36] J. P. Perdew , A. Ruzsinszky , G. I. Csonka , O. A. Vydrov , G. E. Scuseria , L. A. Constantin , X. Zhou , K. Burke , *arXiv preprint arXiv:0707.2088* 2007.

