# Supplementary material for: Metastable Crystalline Cobalt Iron Oxide Nano‐Flakes with Antiferromagnetic/Ferrimagnetic Composition Mosaicity
Source: Angew Chem Int Ed Engl. 2025 Oct 21;64(48):e202504171. doi: 10.1002/anie.202504171 (PMC12643331; doi:10.1002/anie.202504171)
Supplement: Supplementary file 1 — Supporting Information [file ANIE-64-e202504171-s001.pdf]

Supporting Information

Metastable Crystalline Cobalt Iron Oxide Nano-flakes with Antiferromagnetic/Ferrimagnetic Composition Mosaicity

Anna Rabe, Franz-Philipp Schmidt, Shohreh Rafiezadeh, Soma Salamon, Joachim Landers, Mirco Eckhardt, Christoph Pratsch, Benedikt Beckmann, Felix Thomas Haase, David Kordus, Mauricio Lopez Luna, Clara Rettenmaier, Thomas Götsch, G. Wilma Busser, Martin Muhler, Axel Knop-Gericke, Arno Bergmann, Janis Timoshenko, Beatriz Roldan Cuenya, Oliver Gutfleisch, Mirijam Zobel, Rossitza Pentcheva, Heiko Wende, Thomas Lunkenbein, Malte Behrens

Experimental

Synthesis of the three investigated samples was done employing an earlier reported crystalline precursor decomposition approach.[1] The layered double hydroxide precursor was precipitated in an automatic lab reactor system (*OptiMax, Mettler Toledo*) under constant pH and temperature. 125 mL of a metal salt solution containing 0.566 M $Co(NO_3)_2x6H_2O$ and 0.233 M $Fe(NO_3)_3x9H_2O$ was gravimetrically dosed into a single walled glass reactor, prefilled with 200 mL desalinated water, over the course of one hour. A 0.9 M NaOH and 0.06 M Na_2CO_3 basic solution serving as precipitation agent was dosed automatically into the reactor, constantly keeping a pH of 8.5 and the temperature at 50 °C during the precipitation period. The pH was controlled by a Semi-Micro L pH electrode. After precipitation the precipitate was aged for 1 h at 50 °C without further pH control. After cooling down to room temperature, the precipitate was separated from the mother liquor by centrifugation and washed with desalinated water until the conductivity of the washing water fell under 100 µS. The washed powder was dried in static air at 80 °C for at least 12 h.

The as-prepared layered double hydroxide precursor was subjected to thermal treatments in a muffle furnace (*Nabertherm LE 6/11/B150*). With a heating ramp of 2 K min^{-1} thermal treatment was conducted at 400 °C to 900 °C in 50 °C steps, holding times being 3 h up to 850 °C and 17 h for the 900 °C sample.

X-ray diffraction patterns of the samples were recorded on a Bruker D8 Advance with a Cu X-ray source in Bragg-Brentano geometry, using a LYNXEYE XE-T detector. The samples were dispersed in ethanol on a PMMA sample holder and measurements were performed in the range from 5° to 90° 2θ with a step size of 0.01° and a counting time of 1.5 s. For Rietveld refinements for phase analysis, as well as for the determination of the lattice parameters and crystallite size, the TOPAS software was used.

Thermogravimetric analysis of the precursor was carried out on a Netzsch STA449F3A-1226-M thermobalance. The precursor was heated with a rate of β= 2 K min^{-1} in air up to 1000 °C and the mass loss was recorded.

Elemental analysis was performed via inductively coupled plasma – optical spectrometry (ICP_OES, Vista RL; Varian), the O content was determined with the carrier gas hot extraction method (THC 600, LECO).

Scanning electron microscopy (SEM) was conducted with an Apreo S LoVac (Thermo Fisher Scientific). Prior to the measurements, the samples were sputtered with Pt/Au.

Surface area determination for the LDH precursor and samples calcined at 400 °C and 800 °C were carried out in a NOVA 300 (Quantachrome GmbH). 100 mg of each sample were degassed and dried for 2 h at 80 °C in vacuum prior to the measurement. Complete sorption isotherms were obtained at liquid nitrogen temperature.

The surface area of the sample calcined at 900 °C was determined with Kr in a Belsorp-mini setup (MicrotracBEL Corp.) after pretreatment in a vacuum at 150 °C for 24 h.

Powder X-ray diffraction (XRD) for PDF analysis was carried out at room temperature with a STOE STADI P Mythen2 4K diffractometer (Ge(111) monochromator; Ag $K\alpha_1$ radiation, λ = 0.5594 Å) using four Dectris MYTHEN2 R 1K detectors in Debye–Scherrer geometry. Samples were measured in 0.5 mm diameter glass capillaries purchased from Hilgenberg (special purpose glass number 10) for 20 h. The Q-range was 20.4 $Å^{-1}$. For more information on this dedicated diffractometer for pair distribution function analysis, see [2]. PDF processing was carried out with xPDFsuite[3] using a Qmax of 19.5 $Å^{-1}$, and the refinement was done in PDFgui.[4] Parameters refined were the scale, cell parameters, crystalline domain sizes,

correlated atomic motion and atomic displacement parameters. PDF were fit in the range of 1 – 50 Å for the 400 and 800 °C samples, and over 1 – 100 Å for the 900 °C sample.

Scanning transmission electron microscopy in combination with energy-dispersive X-ray spectroscopy (STEM-EDX) was performed on a Thermo Fisher Talos F200X. The microscope was equipped with a high brightness field emission gun (X-FEG) and 4 SDD EDX detectors, giving together a detection area of 0.9 sr. The electron beam energy was 200 keV and the beam current ranged from 100 to 450 pA. The point resolution of the microscope was 1.6 Å. A multiple frame approach was applied to reduce electron beam induced artifacts on the sample. Therefore, the electron beam was scanned across the region of interest several times (up to 210 frames), with short acquisition times ranging from 50 to 100 µs per pixel, and the signal was integrated later. To compensate for sample drift during EDS acquisition, Velox drift correction was applied by cross-correlation after each frame. For EDX quantification, an empirical background model together with Brown-Powell cross sections have been used, as implemented in Velox software. High-resolution transmission electron micrographs (HR-TEM) have been recorded in double-corrected Jeol JEM-ARM200F equipped with a cold field emission gun, operated at 200 keV. TEM images were acquired with a OneView camera by 4kx4k pixels. To minimize for lateral drift, a set of 20 images was recorded with short acquisition times and summed later. To correct for drift and reduce noise, the following post-processing routine was developed and applied. For the post processing we developed an algorithm that suppresses noise in the data by averaging over structures that are similar. In this regard it is similar to Non-Local Means[5] and block matching[6] algorithms but instead of the usual way of finding patches for the averaging a combination of global pattern matching via normalized cross correlation and k-mean clustering is used. This allows for using the information contained in all acquired frames to suppress noise in each individual frame.

Mössbauer spectra were recorded in standard transmission geometry, using a $^{57}Co(Rh)$ radiation source mounted on a constant-acceleration driving unit (WissEl GmbH). Low temperatures and high magnetic fields were achieved with the help of two liquid helium bath cryostats (Thor Cryogenics, Oxford Instruments) containing superconducting split-pair field coils. Data evaluation was carried out with the “Pi” program package, with all spectra being referenced to α-Fe at room temperature.

Magnetic characterization of the whole calcination sample series was carried out with the vibrating sample magnetometer (VSM) option of a Quantum Design PPMS DynaCool, with field dependent M(H) loops being recorded up to maximum fields of ±9 T and temperatures down to 4.3 K. To check for the potential presence of exchange bias effects, the following procedure was utilized: The samples were heated up to 600 K for a duration of approximately 10 min, followed by cooling under selected applied magnetic fields down to the respective measurement temperatures.
For the 400 °C sample, the exchange bias was investigated with the VSM option of a Quantum Design PPMS with M(H) loops subsequent to field cooling in -14 T and +14 T.
For all magnetic measurements the sample powder was filled into a piston-cylinder type sample holder (polypropylene) as supplied by the device manufacturer. This was to ensure a proper compacting of the powder during the measurement in order to avoid any mechanical motion of sample material.

X-ray absorption spectroscopy measurements were performed in transmission mode at the beamline 2-2 at the Stanford Synchrotron Radiation Lightsource (SSRL). A Si(220) monochromator, detuned by 30% was used for energy selection. The intensity of the incident and transmitted X-rays were recorded using 15 cm-long ionization chambers filled with N_2. The beam size was 0.5x5 mm^2. A Co foil was used as a reference sample for data alignment.

XANES data extractions was carried using Athena[7] code.[8] Cobalt and iron oxidation states were calculated from the edge positions from defined reference compounds.

Soft X-ray near-edge X-ray absorption fine structure spectroscopy (NEXAFS) measurements were conducted at the UE56/2-PGM beamline at BESSY II. Auger electron yield (AEY) was measured using a differentially pumped Specs Phoibos 150 hemispherical sector analyzer set to kinetic energies of 560 (Fe-L edge) or 630 eV (Co L-edge). Total electron yield was recorded as the current hitting the spectrometer entrance aperture. All measurements were conducted in high vacuum below 10^{-7} hPa.

The density functional theory (DFT) calculations were performed using Vienna ab initio simulation package (VASP code),[9] employing the projector augmented wave (PAW) method.[10] For the exchange-correlation functional the generalized-gradient approximation in the parametrization of Perdew-Burke-Enzerhof for solids (PBEsol) was used. Static correlation effects are taken into account by applying an on-site Coulomb repulsion parameter U=3 eV on the Co and Fe 3*d* states within the rotationally invariant formulation of Dudarev et al.[11] In this study we modeled the bulk compounds $CoFe_2O_4$, Co_3O_4 and Co_2FeO_4 with different cation and spin arrangements, as well as $Co_3O_4/CoFe_2O_4$ heterostructures with (001) and (111) orientation and for the latter case interfaces along the (111) and ($\overline{11}2$) direction, containing 56, 84 and 168 atoms in the unit cell, respectively.

We used a plane-wave cut-off energy of 500 eV and a Gamma-centered *k*-point mesh of 10 × 10 × 5 for the (111)-oriented bulk of $CoFe_2O_4$, Co_2FeO_4 and Co_3O_4 ,2 × 10 × 5 and 10 x 10 x 2 for the (111) heterostructure along ($\overline{11}2$) and (111), and 10 x 10 x 4 for the (001) heterostructure. Both volume and internal parameters were optimized with the residual forces smaller than 0.01 eV/Å.

Raman spectra were obtained using a LabRAM HR Evolution spectrometer (Horiba) equipped with a confocal microscope containing a 100 times magnification objective. The measurements were performed at room temperature using a green laser (Oxxius, 532 nm, max. 100 mW), applying 1 mW laser power for 600 s with 3 accumulations.

Experiments with increasing laser power were sequentially performed at the same spot, thus applying 1mW when recording a spectrum after dosing resp. 10 mW (60 s), 25 mW (24 s) and 50 mW (12 s).

The method involving increasing the laser power is described by Beatriz Rivas-Murias and coworkers for CoO-Co3O4[12]

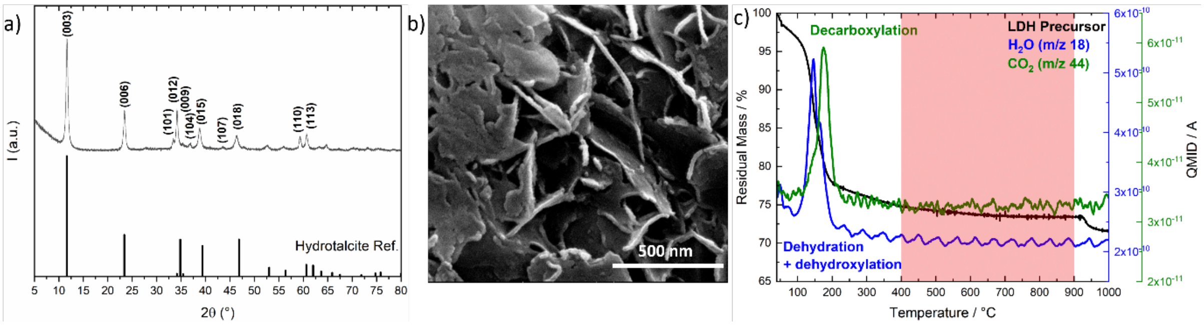

Figure S 1: Characterization data of the $(Co^{2+}_{0.67}Fe^{3+}_{0.33})(OH)_2(CO_3)_{0.17}$ × mH_2O precursor: a) XRD pattern of the as-synthesized LDH precursor, b) SEM micrograph of the as-synthesized precursor, c) and thermogravimetric analysis.

Table S 1: Rietveld refinement parameter for the whole calcination series.

T_{calc} (°C)	R_{exp}	R_{wp}	R_p	R_{exp}'	R_{wp}'	R_p'	GOF
**400**	5.46	6.66	5.25	7.46	9.09	7.51	1.22
**450**	10.58	11.01	8.57	9.79	10.20	8.65	1.04
**500**	10.40	10.81	8.42	9.59	9.97	8.43	1.04
**550**	10.57	10.96	8.49	9.75	10.11	8.56	1.04
**600**	10.50	11.23	8.80	9.83	10.52	8.96	1.07
**650**	10.99	11.48	8.93	9.98	10.42	8.98	1.04
**700**	11.13	12.08	9.38	9.69	10.52	8.95	1.09
**750**	10.64	11.82	9.20	9.59	10.65	9.15	1.11
**800**	9.82	12.14	9.46	9.40	11.62	9.92	1.24
**900**	7.68	12.32	9.70	10.25	16.44	14.07	1.60

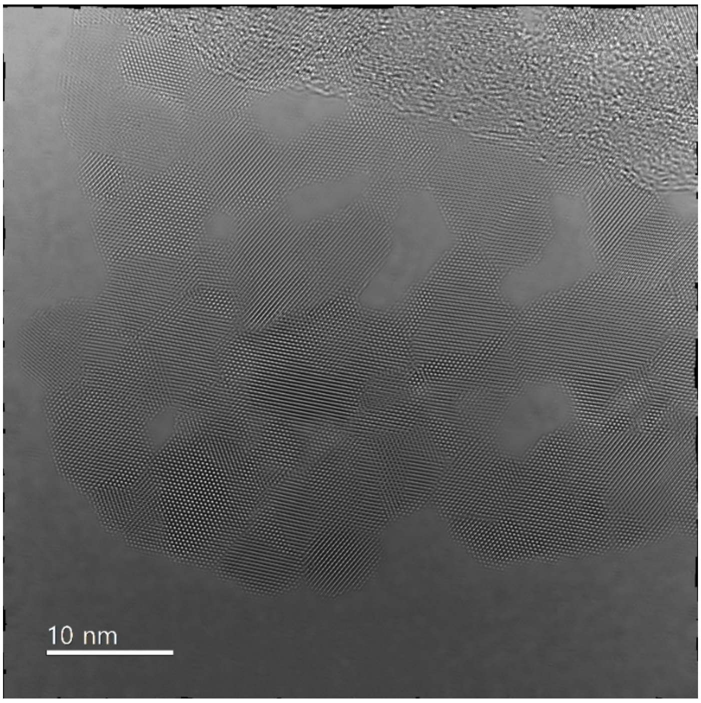

Figure S 2: HR-TEM micrograph of a single Co_2FeO_4 nano flake calcined at 400 °C.

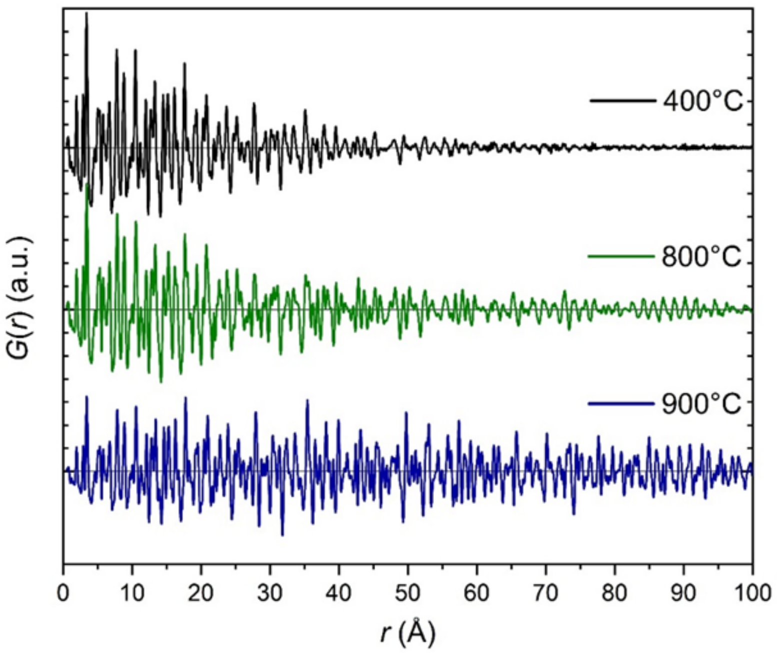

Figure S 3: Experimental PDF data of Co/Fe LDH calcined at 400, 800 and 900 °C displayed up to 100 Å to show the differing crystallinity of the samples.

Table S 2: Results of PDF refinements: refined parameters.

Sample	400 °C	400 °C		800 °C		900 °C
**applied phases**	Co_2FeO_4	Co_3O_4	$CoFe_2O_4$	Co_3O_4	$CoFe_2O_4$	Co_2FeO_4
**ISCD number**	98551	9362	109044	9362	109044	98551
**a (Å) (start)**	8.242	8.065	8.394	8.065	8.394	8.242
**a (Å)**	8.178	8.156(0)	8.208(5)	8.186(1)	8.322(2)	8.247(4)
**crystallite size (Å)**	60	69	65	139	128	“bulk” [a]
$U_{iso,TL}$ **(Å²)**	0.013	0.010	0.013	0.009	0.014	0.011
$U_{iso,TL2}$ **(Å²)**	-	0.010	-	0.011	-	-
$U_{iso,OL}$ **(Å²)**	0.010	0.007	0.014	0.010	0.027	0.009
$U_{iso,O}$ **(Å²)**	0.026	0.015	0.033	0.027	0.045	0.031
**phase ratio**	-	41 %	59 %	50 %	50 %	-
**Fit range (Å)**	1 – 50	1 - 50	1 - 50	1 - 100	1 - 100	1 - 100
R_w	0.20	0.16		0.21		0.18

Discussion of the decreased lattice parameter:

One might argue that the unexpectedly small lattice parameter is caused by a variation in bulk composition, an incomplete transformation of the LDH precursor, or another side phase being present after thermal treatment at 400 °C. To address these possible discrepancies, elemental analyses for determination of the average composition for all three representative samples were performed, the results are shown in Table S 3.

Table S 3: Atomic percentage of cobalt, iron, and oxygen and the respective ratios for the three calcination temperatures.

T_{calc}	Co %at	Fe %at	O %at	Co:Fe:O
**400 °C**	27.2	13.4	59.4	2.03:1:4.43
**800 °C**	29.5	14.5	56.0	2.04:1:3.86
**900 °C**	28.9	14.2	56.9	2.03:1:4.01

Next to the already confirmed ratio of Co:Fe of 2:1 from EDX measurements, the results only show minor deviations from the theoretical ratio for Co:Fe:O of 2:1:4. The ratio of Co:Fe in all cases again is very close to the desired value of 2:1, ruling out the argument of a variation in

bulk composition, and with that eliminating the possibility of an iron deficiency in the sample lowering the overall lattice parameter. An incomplete transformation of the precursor LDH can be ruled out by thermogravimetric analysis and X-ray diffraction, which showed no significant mass loss after 400 °C (1.3%wt between 400 °C and 800 °C) and no crystalline by-phases in the diffraction pattern, respectively. However, small deviations in the overall amount of oxygen are observed in the elemental analysis. The 400 °C sample exhibits the highest amount of oxygen, namely 2.3 at% more than the anticipated 57.1 at% nominal oxygen content. Again, an amorphous by-phase could be ruled out with the help of lattice parameter calculation using Vegard's law. The 2.3 at% oxygen is assumed to be incorporated in an iron containing by-phase. Even if from this it is generously presumed that 3%at iron is missing in the iron rich phase, the resulting lattice parameter is still significantly too small. After ruling out the above-mentioned possibilities, the increased amount of oxygen hints towards a spinel with excess oxygen $Co_2FeO_{4+\delta}$. It is known from literature that super stoichiometric spinels can exhibit decreased lattice parameters.[13] Nevertheless, the decrease in lattice parameter for the iron rich phase in the 400 °C sample is so pronounced that it is concluded that additional effects have to be considered in this case.

DFT:

To gain insight in the experimentally observed phase separation of Co_2FeO_4 in Co- and Fe rich regions and to investigate the origin of the decreased lattice parameter of the iron rich phase, density functional theory (DFT) calculations were performed with the VASP code and PBEsol exchange correlation functional[14] within the generalized-gradient approximation and an additional on-site Hubbard U = 3 eV term on both Co and Fe 3*d* states. We considered the bulk phases of Co_2FeO_4, the end members Co_3O_4 and $CoFe_2O_4$, as well as interfaces between the end members Co_3O_4 and $CoFe_2O_4$. In particular, we have considered interfaces with (111) and (001) surface orientation. For the (111)-orientation both interfaces along the c- and the a-axis were modelled, the latter being closest to the experimental observation of (111)-oriented platelets with mosaic pattern of Co- and Fe-rich regions. In the normal spinel Co_3O_4, low spin (LS) Co^{3+} cations with a quenched magnetic moment (see the spin density in Figure S 4) occupy the octahedral sites, whereas Co^{2+} cations with a magnetic moment of 2.6 μ_B fill the tetrahedral sites. The latter order antiferromagnetically, rendering Co_3O_4 an antiferromagnetic semiconductor. The calculated bulk lattice constant of 8.01 Å is 0.6-0.8% smaller than the experimentally reported value of 8.06-8.08 Å[15] and in agreement with previous theoretical values of 8.04 Å (PBEsol+U, U = 4.4 eV).[16]

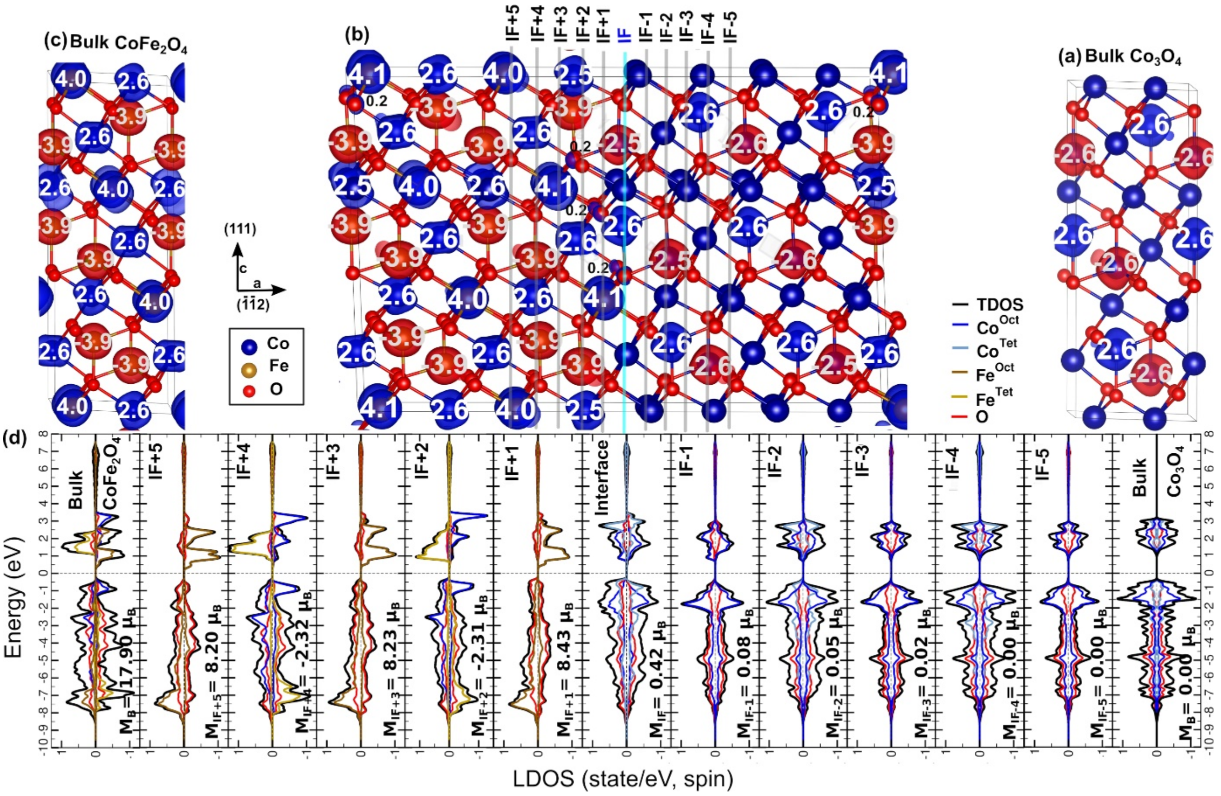

Figure S 4: Structure and spin density of (111)-oriented (a) bulk Co_3O_4, (b) heterostructure of $CoFe_2O_4$ and Co_3O_4 with an interface, perpendicular to the a direction and (c) bulk $CoFe_2O_4$. Blue and red colors represent the majority and minority spin density, respectively. (d) Layer and element-resolved density of states of the Co_3O_4/$CoFe_2O_4$(111) heterostructure and also bulk of Co_3O_4 (right) and $CoFe_2O_4$ (left). M_{IF-n} presents the total magnetic moment of each layer at heterostructure in μ_B. The color code for the partial LDOS contribution of different ions is indicated in the upper right of LDOS.

On the other hand, in the fully inverse $CoFe_2O_4$ spinel, Co^{2+} cations with a magnetic moment of 2.6 μ_B occupy the octahedral sites, whereas the Fe^{3+} cations fill both, tetrahedral and octahedral sites with magnetic moments of -3.9 μ_B and 4.0 μ_B, respectively. The latter two couple antiferromagnetically; the parallelly aligned Co^{2+} moments lead to the ferrimagnetic behavior of $CoFe_2O_4$. The calculated lattice constant of 8.33 Å is close to the experimental values (8.37-8.39 Å).[17] Moreover, the bulk modulus of $CoFe_2O_4$ is considerably smaller (187.7 GPa) than the one of Co_3O_4 (232.9 GPa), indicating a stronger compressibility.

For Co_2FeO_4 with the inverse spinel structure, Co cations are distributed at the tetrahedral and octahedral sites with a magnetic moment of 2.5 μ_B (Co^{2+}) and 0.0 μ_B (Co^{3+}), respectively. Fe^{3+} cations are located at octahedral sites with magnetic moments of 4.1 μ_B. The calculated lattice constant of 8.19 Å is 0.6% less than the experimental value of 8.24 Å.[15b, 18] (see also present XRD and PDF measurements presented in Table 1 in the main text). Previous results using PBE+U (U_{Co}= 2 eV, U_{Fe}= 3 eV) [19] render a higher value of 8.27 Å As presented in Table S 4, the calculated bulk modulus of 238 GPa for Co_2FeO_4 is higher than the value of 187.7 GPa for $CoFe_2O_4$ (experimental value: 185 GPa)[20] and 232.9 GPa for Co_3O_4 (experimental value

249±1 GPa).[21] Additionally, we also calculated the bulk modulus, lattice constant of other possible configurations, e.g. considering low spin states for Fe and Co and normal as well as inverse phases. The energy difference to the most stable phase (ΔE) and the heat of formation (ΔH) are presented in Table S 4.

Table S 4: Calculated lattice constants and bulk modulus for $CoFe_2O_4$, Co_3O_4 and Co_2FeO_4 for both normal and inverse spinel structures and considering Fe and Co ions for both low and high spin states with PBEsol+U ($U_{Co,Fe}$ = 3 eV). ΔE is the energy difference of each phase from the most stable one. ΔH is the heat of formation.

Structure	**Lattice constant (Å)**	**Bulk modulus (B_0) GPa**	**ΔE (eV/f.u)**	**ΔH (eV)**
inverse – $CoFe_2O_4$	**8.33** (Exp: 8.37-8.39)[17]	**187.7** (Exp: 185)[20]	**0.0**	**-11.43**
normal – $CoFe_2O_4$	**8.37**	**183.8**	**0.25**	**-11.18**
inverse – $CoFe_2O_4$ –Fe: low spin	**8.21**	**363.0**	**2.74**	**-8.70**
inverse – $CoFe_2O_4$ – Fe & Co: low spin	**7.97**	**247.8**	**3.21**	**8.23**
normal – $CoFe_2O_4$ – Fe: low spin	**8.10**	**248.0**	**1.58**	**-9.85**
normal – $CoFe_2O_4$ – Fe & Co: low spin	**8.14**	**561.7**	**2.62**	**-8.82**
normal – Co_3O_4	**8.01** (Exp: 8.06-8.08)[15]	**232.8** (Exp: 249±1)[21]	**0.0**	**-9.78**
inverse – Co_2FeO_4	**8.19** (Exp: 8.24)[15b, 18, 22]	**238.8**	**0.0**	**-10.32**
inverse – Co_2FeO_4 – Fe: low spin	**8.06**	**252.5**	**0.55**	**-9.77**
normal - Co_2FeO_4	**8.10**	**278.8**	**0.45**	**-9.87**
normal – Co_2FeO_4 – Fe: low spin	**8.04**	**549.7**	**0.86**	**-9.45**

According to the HR-TEM images, the platelets are oriented along the (111)-direction with Co and Fe rich regions in a mosaic pattern. To model as close as possible to the experimental observation we have considered explicitly heterostructures of (111) oriented $CoFe_2O_4$ and Co_3O_4 with a lateral interface along the hexagonal *a*-direction (Figure S 4). The DFT+*U* results for further heterostructures with (001) and (111) oriented interfaces are shown in Figure S 5.

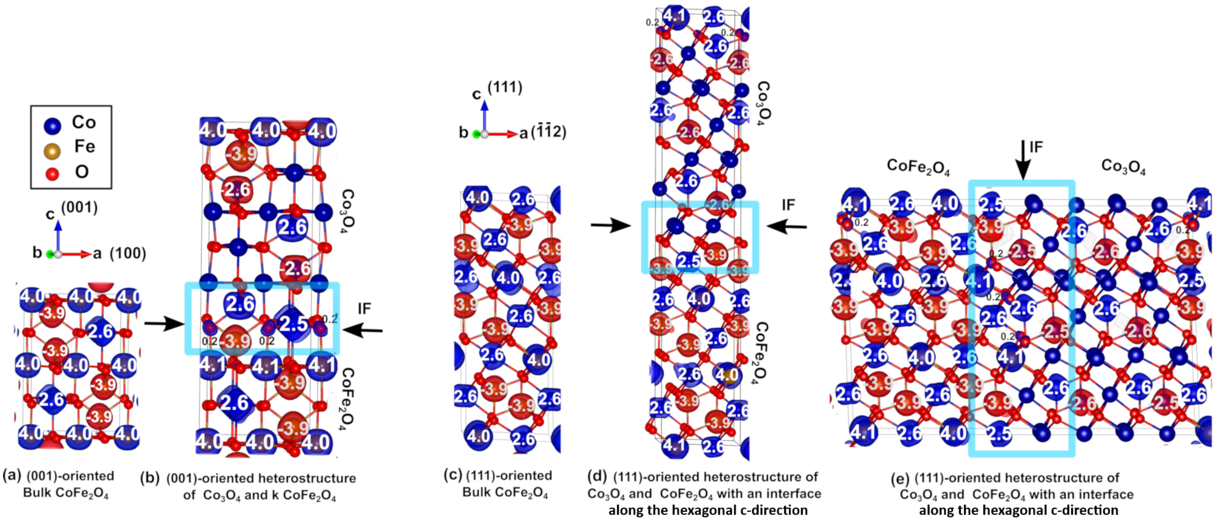

Figure S 5: Side view of the structure and spin density of bulk phases and heterostructures with different orientations: (a) (001) and (c) (111) oriented bulk of $CoFe_2O_4$ (b) (001) and (d) (111)- oriented heterostructure of $CoFe_2O_4$ and Co_3O_4 with an interface along the (d) (111) (e) ($\bar{1}\bar{1}2$) direction.

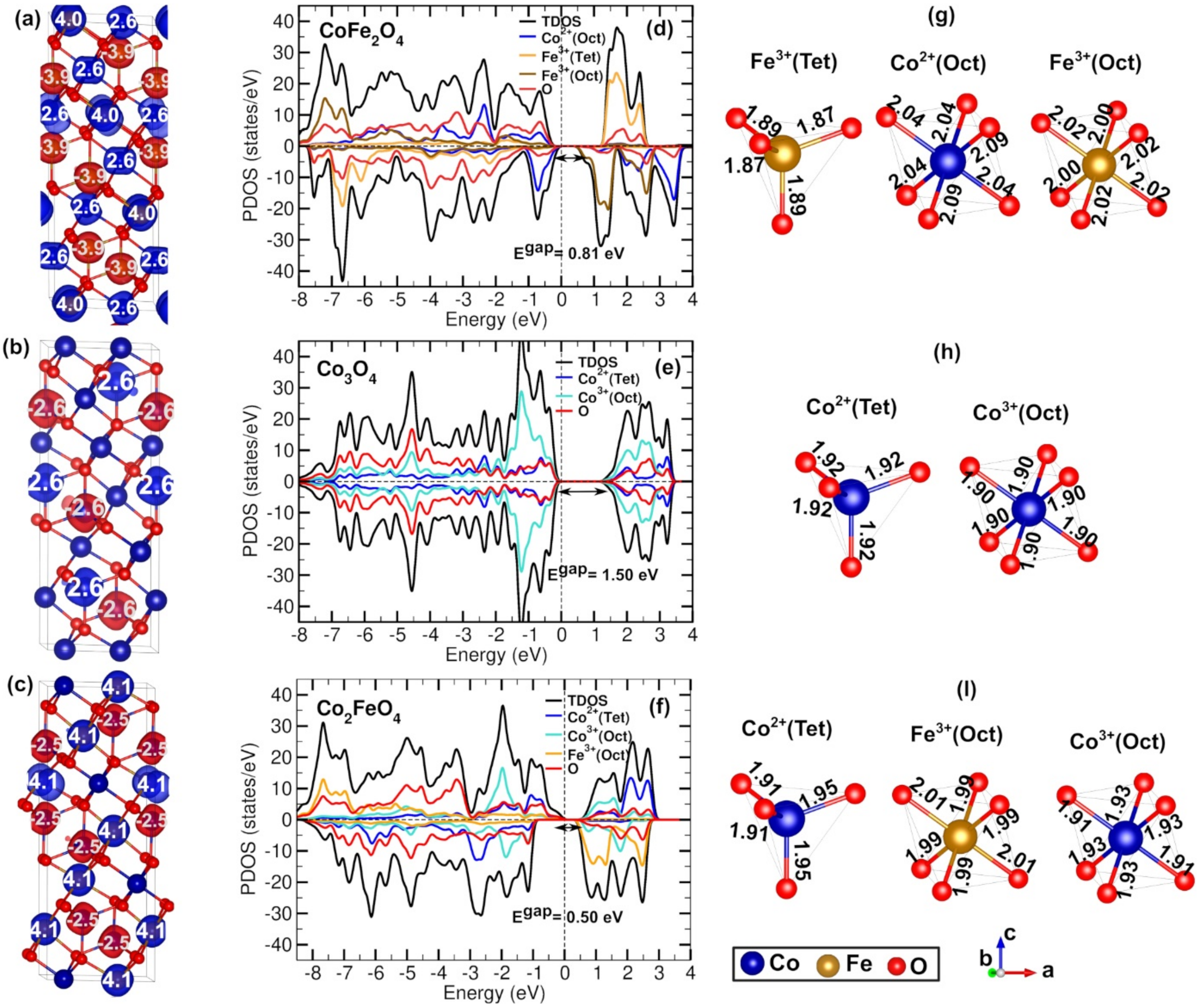

Figure S 6: The spin density of bulk (a) $CoFe_2O_4$, (b) Co_3O_4, and (c) Co_2FeO_4 with their corresponding projected density of states (d,e,f,). The respective Fe-O and Co-O bond length at the tetrahedral (Tet) and octahedral (Oct) sites are presented in g-l.

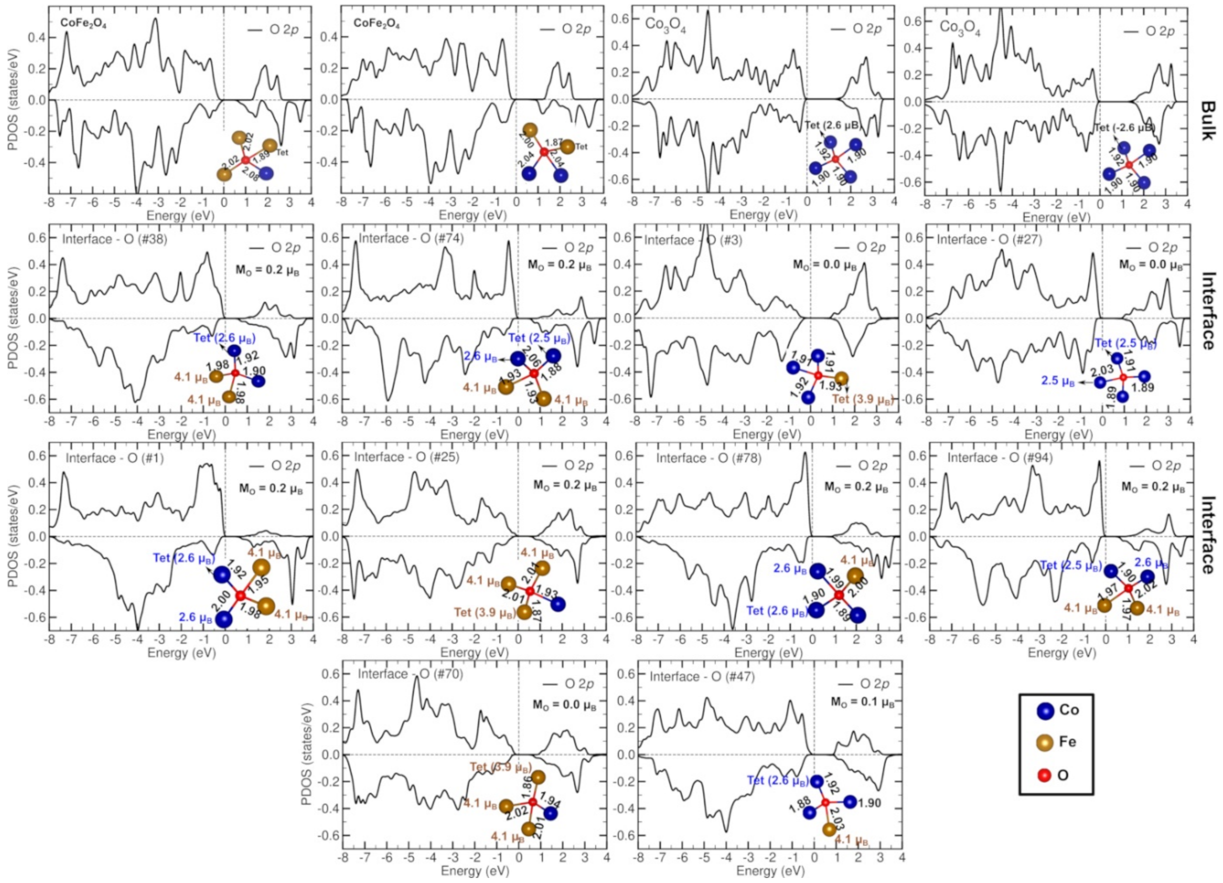

Figure S 7: Projected density of states (PDOS) of oxygen 2p states in the bulk end members (first row) and the at the interface of the Co_3O_4/$CoFe_2O_4$(111) heterostructure. The positions of the oxygen ions are numbered according to Figure S 8. Tetrahedrally coordinated cations are denoted by Tet, the remaining are cations are positioned at the octahedral sites. All reported bond length (black) are in Å. M_o represents the magnetic moment of oxygen at the interface.

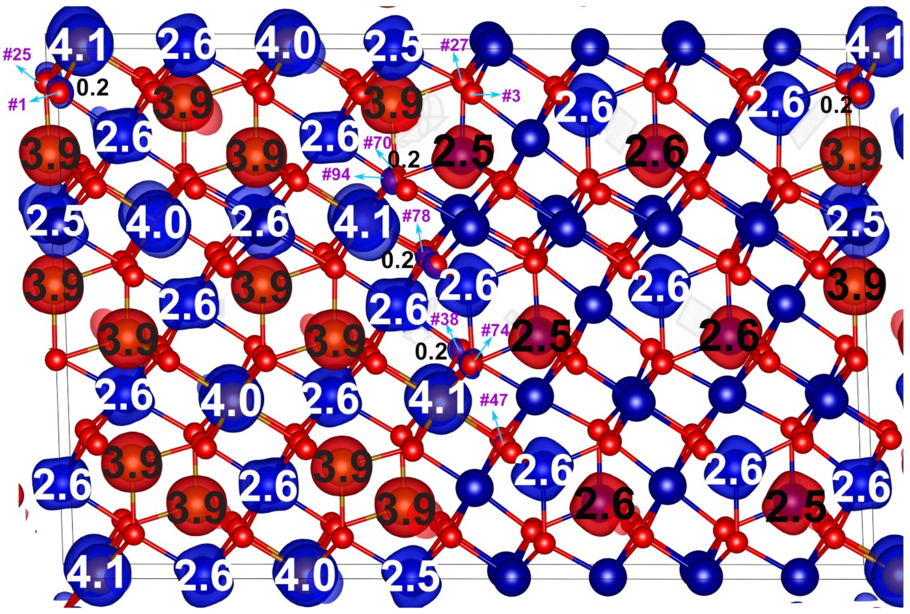

Figure S 8: Side view and spin density of Co_3O_4/$CoFe_2O_4$(111) heterostructure with an interface along the (112) direction. #n indicates the oxygens number at the interface of Co_3O_4 and $CoFe_2O_4$.

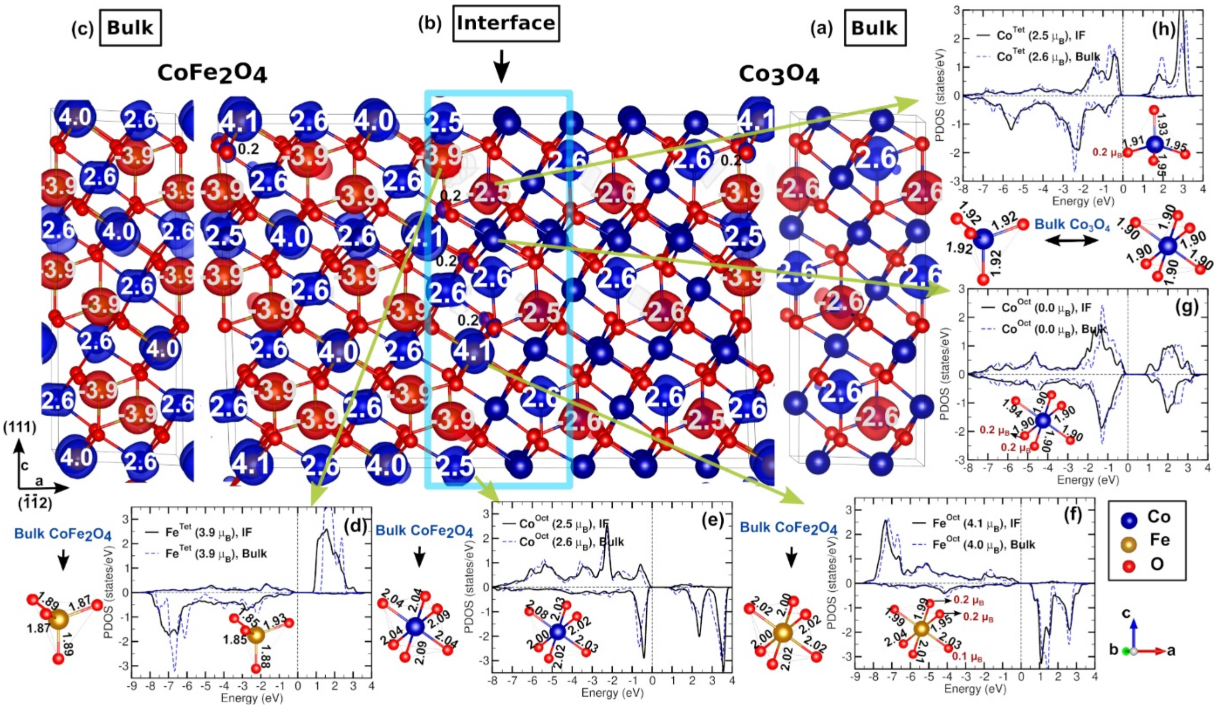

Figure S 9: Structure and spin density of (111)-oriented (a) bulk $CoFe_2O_4$, (b) heterostructure of $CoFe_2O_4$ and Co_3O_4 with an interface, perpendicular to the a direction and (c) bulk Co_3O_4. Blue and red colors represent the majority and minority spin density, respectively. Additionally, the projected density of states (PDOS) of cations at the interface: (d) Co^{tet}, (e) Co^{oct}, (f) Fe^{tet}, (g) Co^{oct}, and (h) Fe^{oct} (black solid lines) is compared to the respective PDOS in the end member (dashed lines). The insets show the local environment and the Co-O and Fe-O bond length in Å for cations at the interface and the respective bulk end members.

The structural optimization of the $Co_3O_4/CoFe_2O_4$ heterostructure resulted in a decreased volume of 556.63 Å^3, which corresponds to a cubic lattice constant 8.22 Å in the Fe-rich part ($CoFe_2O_4$) of the heterostructure (experiment: 8.171 Å (XRD) and 8.208 Å (PDF)), compared to the bulk volume of 577.13 Å^3 (a = 8.33 Å). The Co-rich part of the heterostructure, Co_3O_4, has a volume of 533.033 Å^3 (a= 8.11 Å) in agreement with measurements (8.129 Å (XRD) and 8.156 Å (PDF)) which shows a slight increase compared to the Co_3O_4 bulk volume of 513.026 Å^3 (a = 8.01 Å) in agreement with the experimental observations. The $Co_3O_4/CoFe_2O_4$(111) heterostructure is found to be 0.15 eV/f.u. more favorable than the initial stoichiometry Co_2FeO_4 which indicates that the separation in the two phases is more favorable than the formation of Co_2FeO_4 in agreement with experiments.

A side view of the $Co_3O_4/CoFe_2O_4$(111) heterostructures together with the spin density is shown in Figure S 4 along with the spin densities of the bulk phases (Figure S 4a and c). While no drastic changes are observed at the cation sites, the magnetic moment of Fe^{3+} at the octahedral sites close to the interface is slightly enhanced to 4.1 μ_B (bulk value: 4.0 μ_B). The magnetic moment of Co^{2+} at the tetrahedral sites close to the interface in the $CoFe_2O_4$ part is reduced to 2.5 μ_B, the remaining Co^{2+} ions having a magnetic moment of 2.6 μ_B, similar to bulk.

Additionally, we observe a noticeable spin-polarization of 0.2 μ_B at oxygen sites close to the interface.

The layer and element-resolved density of states, presented in Figure S 4d gives further insight into the band alignment and electronic reconstruction at the interface. In particular, the band gaps towards the center of each phase resemble the ones of the bulk phases (0.81 eV in $CoFe_2O_4$ and 1.5 eV in Co_3O_4, see Figure S 6 for comparison), but are modified close to the interface, e.g. the one of Co_3O_4 is reduced. Remarkably, a significant exchange splitting in the majority and minority channel is obtained in the interface (IF) Co_3O_4 layer resulting in 0.42 μ_B net magnetization, in contrast to the zero net magnetization of the antiferromagnetic bulk Co_3O_4. The effect fades away from the interface and converges to bulk behavior towards the center of the Co_3O_4 region. A slight enhancement of the total magnetization is also obtained in the interface (IF+1) $CoFe_2O_4$ layer (8.43 μ_B) compared to the IF+3 (8.23 μ_B). The interface-induced magnetization may be responsible for the experimentally observed exchange bias. For further analysis, the projected density of states (PDOS) of Co and Fe cations at the interface as well as the corresponding Fe-O and Co-O bond lengths are plotted and compared to the ones in bulk Co_3O_4 and $CoFe_2O_4$ for both tetrahedral and octahedral sites in Figure S 9d-h. In general, subtle changes occur and a slight downward shift of bands at the interface w.r.t. the bulk PDOS of Co_3O_4 and $CoFe_2O_4$. Overall, the Co^{Tet}-O bond lengths at the interface tend to be longer than in the respective bulk environments, the opposite trend is observed for the octahedral sites and Fe^{Tet}-O bond lengths. In particular shorter Fe^{Oct}-O bond lengths of 1.95 and 1.99 Å compared to the bulk (2.0 and 2.02 Å) emerge at the interface to oxygen ions with magnetic moments of 0.2 μ_B. Such an effect is not observed for the Co^{Oct}-O (Fe-rich part), the oxygen ions remain nonmagnetic in this case. The Co^{Oct}-O bond lengths (Co-rich) do not show remarkable changes compared to the bulk. These subtle changes at the interface between the antiferromagnet (Co_3O_4) and the ferrimagnet ($CoFe_2O_4$) are likely connected to the observed exchange bias – an effect that was previously observed for other mostly polar oxide interfaces, e.g. CoO/Co_3O_4 [23] and Fe_2O_3/$FeTiO_3$.[24]

Mössbauer Spectroscopy:

The sample calcined at 900 °C displays two clearly separated subspectra as shown by the associated Mössbauer spectrum (Figure 7 in the main text and Figure S 10), representing Fe atoms on tetrahedrally coordinated A-sites (blue) and octahedrally coordinated B-sites (green) of the spinel lattice. We observe overall sharp spectral lines, reproduced via narrow hyperfine field distributions corresponding to a low variation of local surroundings of the Fe atoms, strongly suggesting a single-phase state of the sample after calcination in agreement to XRD data. The spectrum displays low intensity of lines 2 and 5 due to a very low degree of spin canting, which can be converted to spin canting angles of ca. 21(1) ° and 0(9) ° for the B- and A-site, respectively.[25] A comparison of the subspectral intensities indicates an occupation of 21(2) % of Fe^{3+} ions on tetrahedral lattice positions, and thereby a preferential B-site placement.[26]

After calcination at 800 °C, the Mössbauer spectrum is accompanied by a slight increase in spin-canting, the spectral component of higher nuclear Zeeman splitting now consists of two subspectra (purple color in Figure 7 in the main text and Figure S 10) with slightly different hyperfine magnetic fields. This effect is more pronounced by comparing spectra of samples annealed at intermediate temperatures in the range of 450-750 °C shown in Figure S 10. Presumably, this is caused by the presence of two distinct phases, where Fe atoms are placed on A-sites in either an Fe-rich ($CoFe_2O_4$-like) or Co-rich (Co_3O_4-like) environment, resulting in a minor variation of the hyperfine magnetic field.[27] However, it should be noted that findings by Takahashi et al indicated that at low temperatures the A sublattice magnetization may be dominant in cobalt rich spinels, in which case the violet subspectrum could correspond to B-site Fe^{3+} in an cobalt rich surrounding, alternatively.[26]

For the 400 °C sample, the degree of spin canting is significantly increased which leads to a partial overlap of the three subspectra and thus individual contributions are difficult to resolve. Enhanced spin canting is indicative for magnetic pinning, which is here likely caused by the high specific interface area (domain sizes of only ca. 5 nm) of the strongly intermixed regions of differing composition. The spin canting increases rather abruptly for lower calcination temperatures, which is explained by the stronger interface coupling of both phases. The Co-rich phase tends to order antiferromagnetically for $x_{Co} \lessapprox 3$, also preventing the full orientation of the adjacent ferrimagnetic Fe-rich ($CoFe_2O_4$-like) phase, which is usually easily aligned at moderate magnetic fields of 10 T.

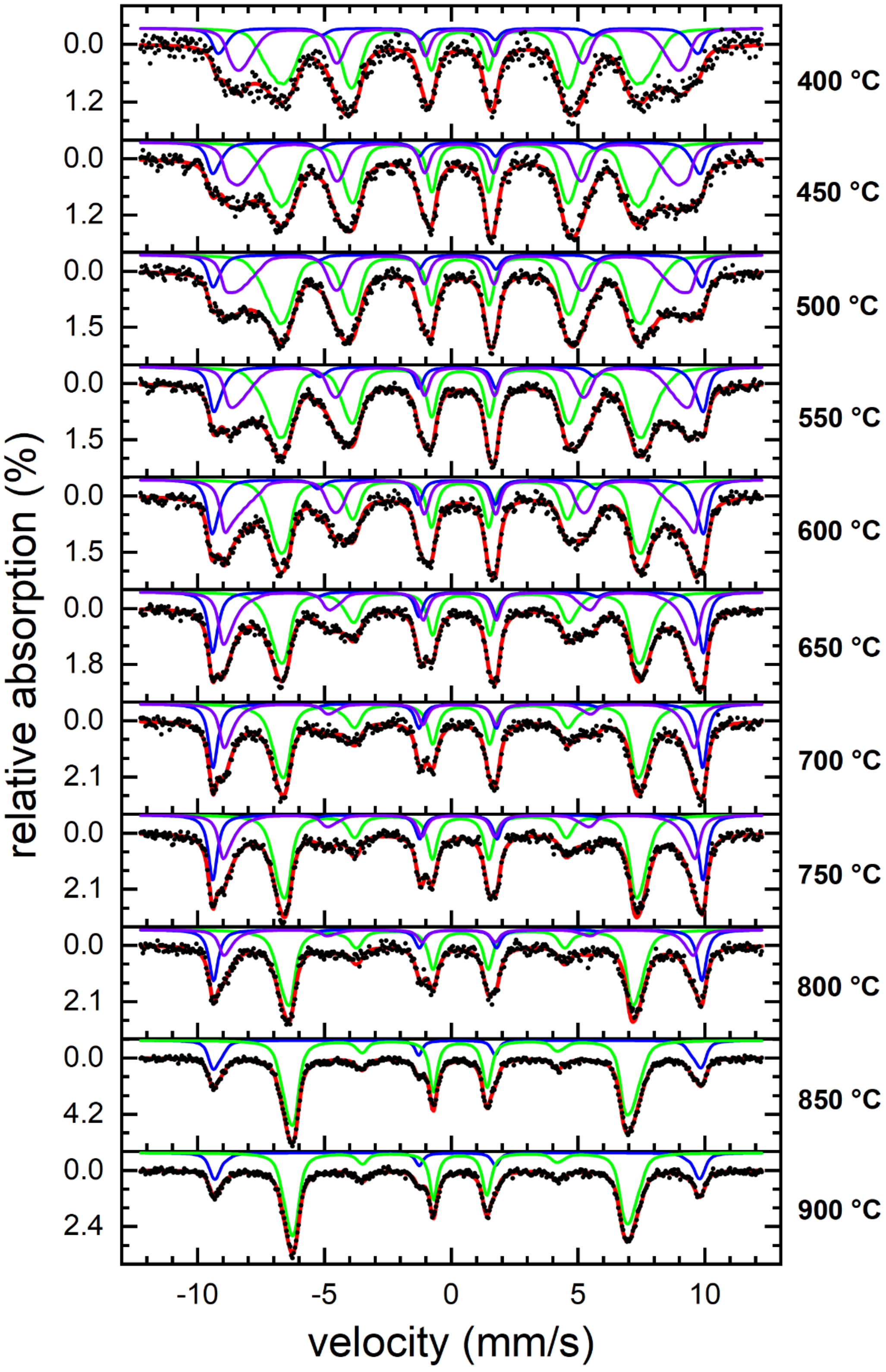

Figure S 10: Mössbauer spectra measured for the complete calcination series in an external magnetic field of 10 T.

Figure S 10 shows the 10 T Mössbauer spectra for the complete calcination series. The spectra of the samples calcined at or below 800 °C show a splitting of the A site contribution into two contributions. It is assumed that the contribution with the lower isomer shift and higher hyper fine field (blue) can be assigned to the iron rich phase and the second contribution (purple) to a phase with a lower iron content.

Additionally, information about the individual phases is now accessible, e.g., the degree of canting. If for example the intensity ratio A_{23} is evaluated, a significantly higher magnetic frustration for the iron depleted phase compared to the iron rich phase is identified. This was anticipated, since the cobalt rich phase exhibits a stoichiometry between iron cobaltite and pure cobalt spinel, which is antiferromagnetic at 5 K. The iron rich phase on the other hand has a composition similar to cobalt ferrite and hence can be easily aligned in a 10 T magnetic field.

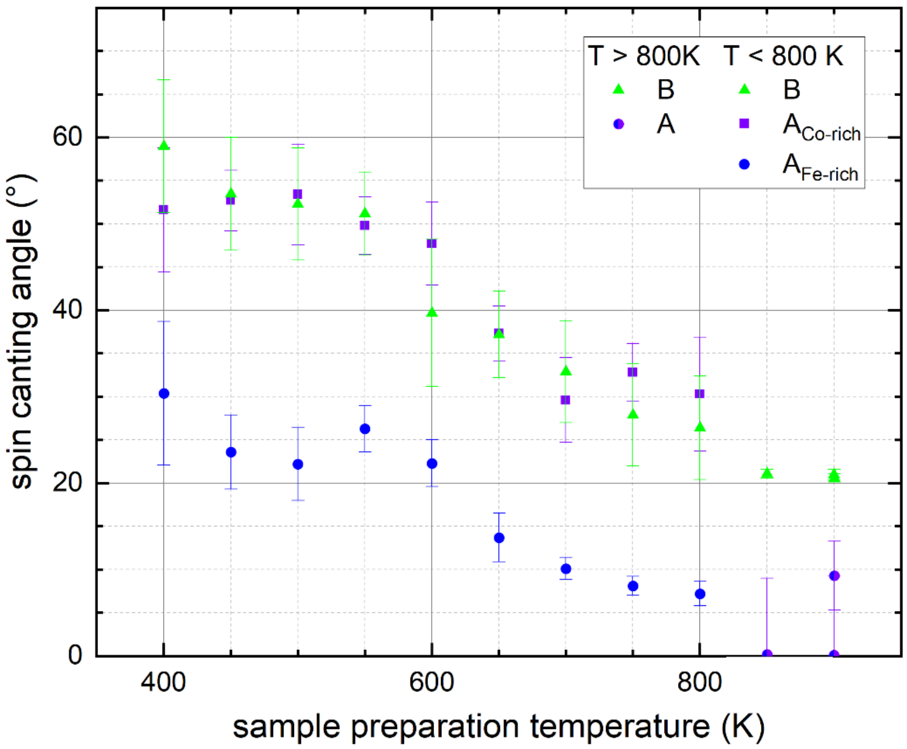

Figure S 11: Intensity ratio A_{23} from Mössbauer spectroscopy of the calcination series.

Magnetometry

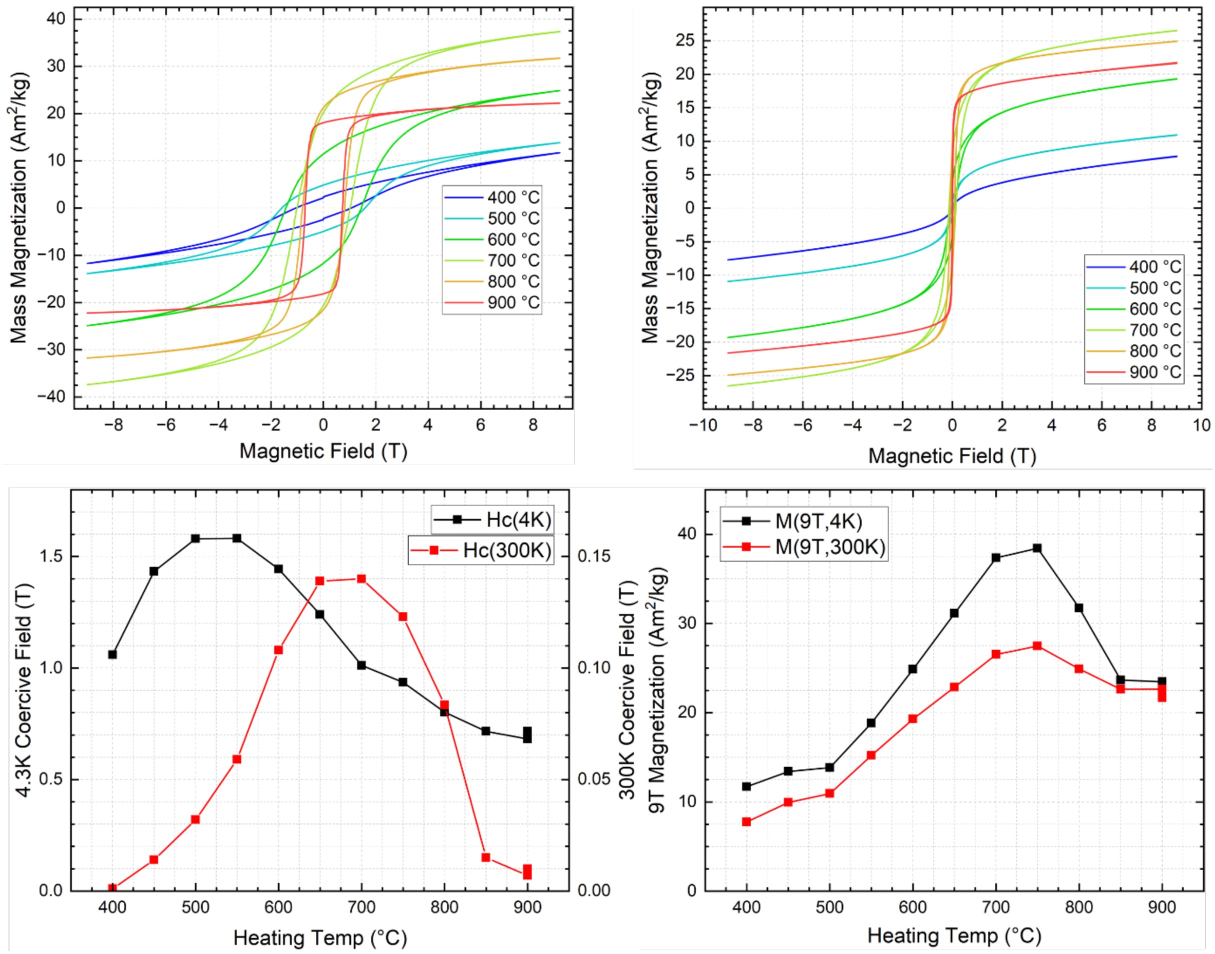

Figure S 12: M(H) 4.3 K and a maximum field of 9 T. b) M(H) at 300 K and a maximum field of 9 T. c) Coercive fields at 4.3K and 300 K. d) High field (9 T) magnetization at 4.3 K and 300 K.

Figure S 12a and b show M(H) curves recorded up to a maximum field of 9 T at 4.3 K and 300 K, respectively, presenting the effect of different calcination temperatures on the magnetic properties. While low calcination temperatures result in an M(H) behavior similar to incompletely compensated antiferromagnet, higher calcination temperatures result in ferrimagnetic behavior, with a rather rectangular shape for maximum calcination temperatures T_{calc} of 900 °C. At 300 K, for low calcination temperatures, we no longer observe open hysteresis loops, which could point to superparamagnetic behavior as discussed below in more detail.

Coercive fields and high field (9 T) magnetization values were extracted from the measurements shown in a) and b), and are depicted in Figure S 12c and d, respectively. One has to keep in mind that the majority of the samples shown here are composed of two distinct phases, with the data chiefly representing the Fe-rich phase of higher coercivity. Considering determined lattice constants (as shown in Figure 5 in the main text) and the limited speed of phase separation at low T_{calc}, it is reasonable to assume that the trend in $H_C(T_{calc})$ is directly

correlated to the composition of the Fe-rich phase (the higher H_C, the more pronounced the phase separation). At 300 K, H_C is strongly reduced for maximum heat treatment, as here, the single phase $FeCo_2O_4$ exhibits a Curie temperature T_C of ca. 400 K. For lower values of T_{calc}, the ordering temperature of the Fe-rich phase is expected to be considerably higher, resulting in a lower reduction in H_C. Decreasing T_{calc} even further leads to monotonous decrease in H_C, reaching close to zero coercive fields for T_{calc} = 400 °C. Presumably, this can be explained by the continuous decrease in size of the Co- and Fe-rich domains with decreasing T_{calc}. As the T_C of the Co-rich phase is expected to already be exceeded at 300 K, the system could be considered as small ferrimagnetic regions in a paramagnetic matrix, whereby the distinct reduction in H_C could be assigned to superparamagnetic relaxation.

9 T magnetization values, used to approximate saturation magnetization behavior, display a marked increase up to ca. 40 Am^2/kg for T_{calc} of 750 °C, when going from single-phase to phase separated materials. Based on estimations of the stoichiometries of both phases, it can be concluded that the Co-rich phase may have a negligible contribution to magnetization, being close to the point of compensation, where A- and B-sublattice magnetization cancel each other out. The relatively high maximum magnetization in this case would stem almost exclusively from the more cobalt-ferrite-like Fe-rich phase.[26] For T_{calc} < 700 °C, M(9 T) continuously decreases, which can be explained by increasing magnetic frustration, as illustrated via in-field Mössbauer spectroscopy, whereby M(9 T) here becomes an increasingly poor approximation of the saturation magnetization.

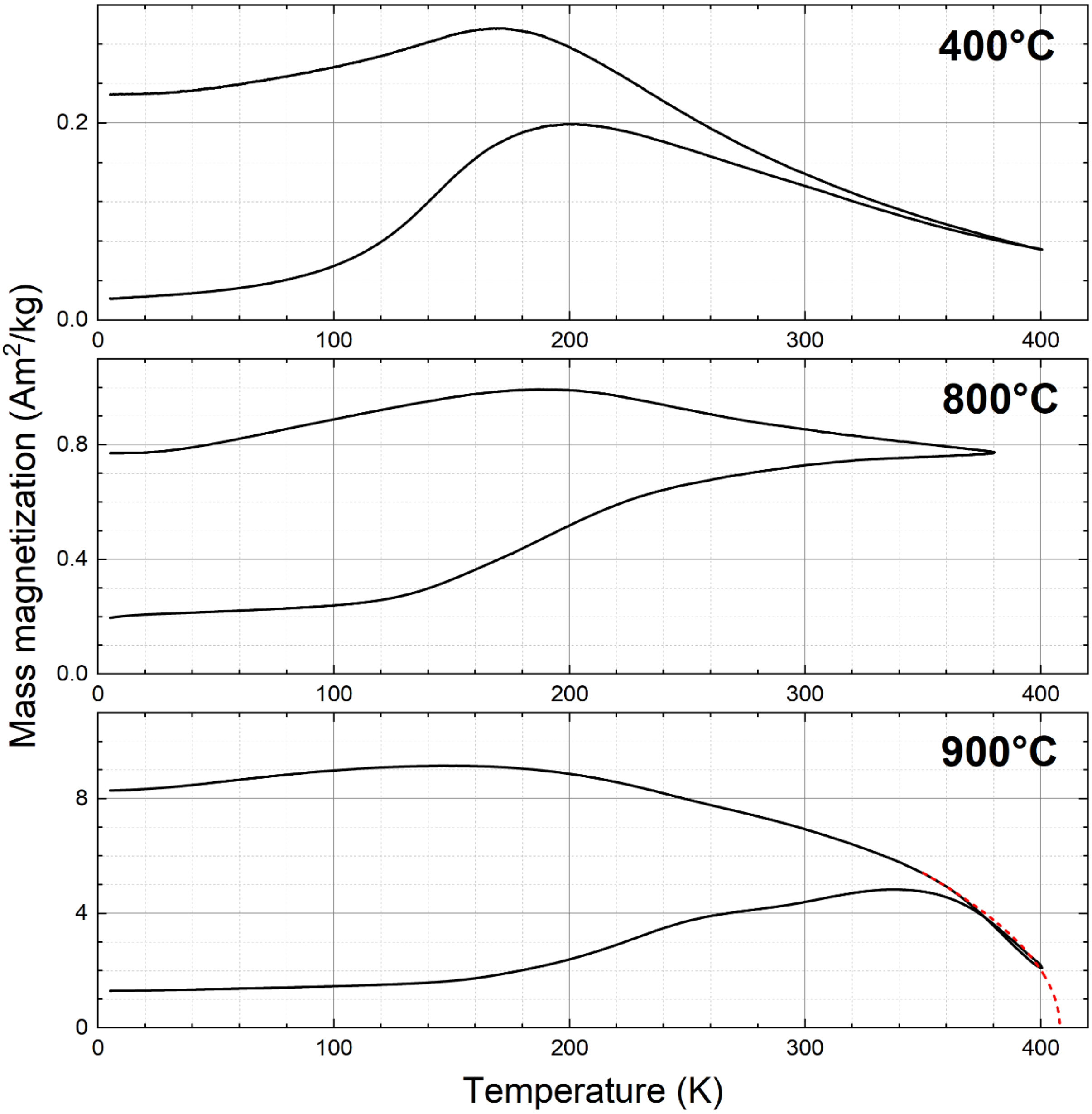

Figure S 13: ZFC-FC magnetization curves of sample material annealed at 400°C, 800°C and 900°C recorded at 5 to 400 K (380 K for the 800 °C sample) in an external magnetic field of 10mT. Red line: Guide to the eye

ZFC-FC magnetization curves may provide valuable information on magnetic sample structure, indicating e.g. Néel- and/or Curie temperatures, being especially relevant for the discussion of exchange-bias effects originating from the coupling of Fe-rich and Fe-poor local regions. The 400 °C sample displays peak features in FC- and ZFC-magnetization closely below 200 K, likely indicating the Néel-temperature of the Fe-poor sample phase, dominating the sample properties. At higher temperatures, ZFC- and FC-magnetization show minor splitting while showing a Curie-Weiss-like shape, at the same time. Similarly, the 800°C sample shows a maximum in ZFC-magnetization at a slightly higher temperature, assigned to the Néel temperature of the Fe-poor phase. When comparing this value of T_N with measurements by Takahashi et al. of quenched Co_2FeO_4 material, we yield stoichiometries in the range of

$Fe_{0.5}Co_{2.5}O_4$ for iron poor phase and $Fe_{1.5}Co_{1.5}O_4$ for the iron rich phase.[26] In agreement with Fe-contents based on EDX experiments and lattice parameters from Rietveld refinement: under the assumption that Vegards law is valid for these samples, by comparing lattice parameters stated in the main text for the 800 °C sample with those of Co_3O_4 and $CoFe_2O_4$, we yield values consistent with above mentioned stoichiometries. At higher temperatures, FC- and ZFC-magnetization remain split, due to the ferrimagnetic properties of the Fe-rich component, exhibiting higher ordering temperatures, for e.g. $Co_{1.5}Fe_{1.5}O_4$ being expected at ca. T_C ~ 700 K. The 900 °C sample does not show pronounced or narrow peak features, instead the magnetization fastly decreases when approaching 400 K. This is consistent with the 900 °C sample being in a homogeneous single-stoichiometric state, with the ordering temperature indicated by the trend in magnetization of T_C ~ 408 K being in fair agreement with values determined by Takahashi et al.[26]

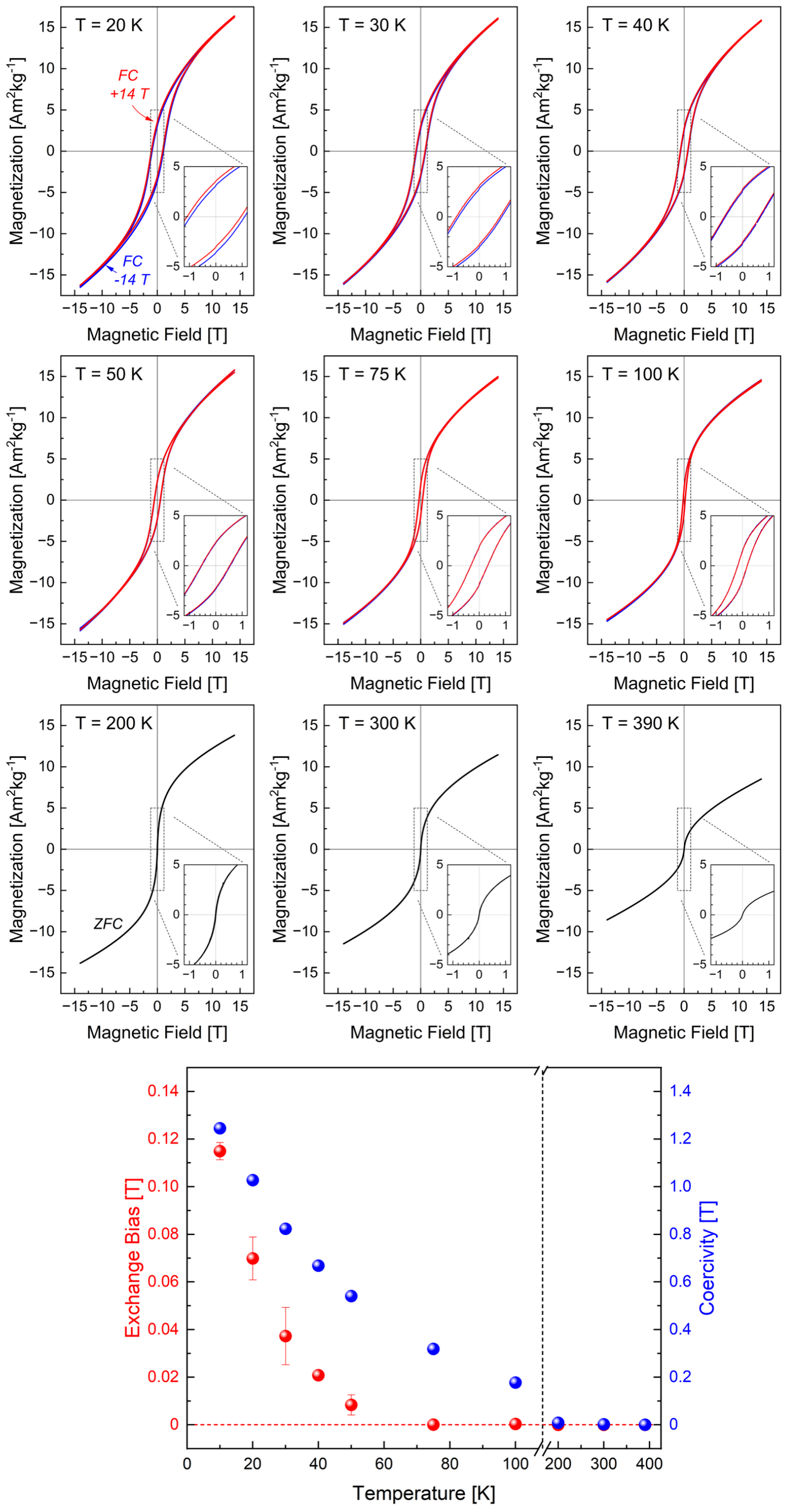

Figure S 14: M(H) curves recorded between 20 K and 390 K after field cooling +- 14 T and the extracted exchange bias and coercivity.

Raman Spectroscopy

Raman spectra (using a 532 nm laser, applying a power of 1 mW) were recorded of the nanoflakes calcined at 400 °C, 800 °C and 900 °C. In Fig S15a, these spectra are displayed and compared to those of the reference materials Co_3O_4 and $CoFe_2O_4$. The spectra contain characteristics of those of Co_3O_4 and $CoFe_2O_4$ but also show distinct differences and cannot be constructed by a summation of both (Fig S15c). The spectra of materials calcined at 400 °C and 800 °C are very similar to those reported for the formation of periodic nanostructures obtained by spinodal decomposition of $Co_{1.7}Fe_{1.3}O_4$ thin films annealed at 600 °C between 12 and 96 h.[28] In those reported materials, cobalt rich ($Co_{2.1}Fe_{0.9}O_4$) and iron-rich ($Co_{1.3}Fe_{1.7}O_4$) regions of about 50 nm in diameter have been identified for the sample annealed after 96 h. These spectra show a strong red shift of the high intensity band compared to the $A_{1g}(2)$ band of CFO and $A_{1g}(1)$ band of Co_3O_4. In the spectrum of the nanoflakes calcined at 400 °C (Fig S15b), the more pronounced appearance of the band at the position of 513 cm^{-1} similar to the F_{2g} band observed for Co_3O_4 (518 cm^{-1}) suggests that the cobalt rich phase has a composition closer to Co_3O_4. With increasing calcination temperature, this band is decreasing (Fig S15a) and the spectrum of the nanoflakes calcined at 900°C more resembles the spectrum reported for $Co_{1.7}Fe_{1.3}O_4$ thin films annealed at short times,[28] shortly before the segregation starts. The composition of that sample is close to a spinel with the composition Co_2FeO_4. We therefore conclude that the spectrum of the nanoflakes calcined at 900 °C is representative of a spectrum of Co_2FeO_4 which is confirmed by XRD and Rietveld refinement.

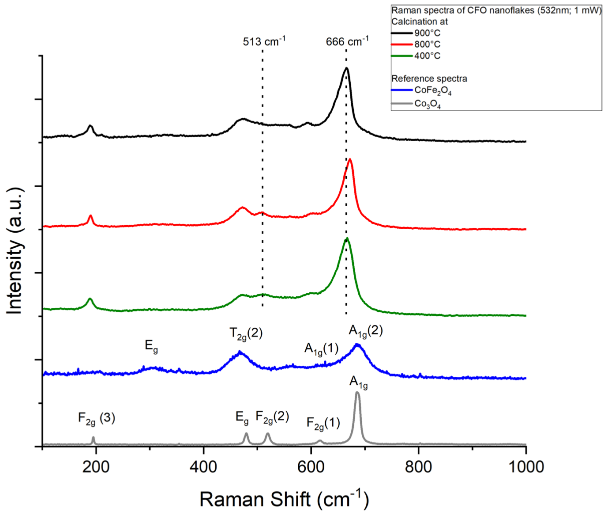

a)

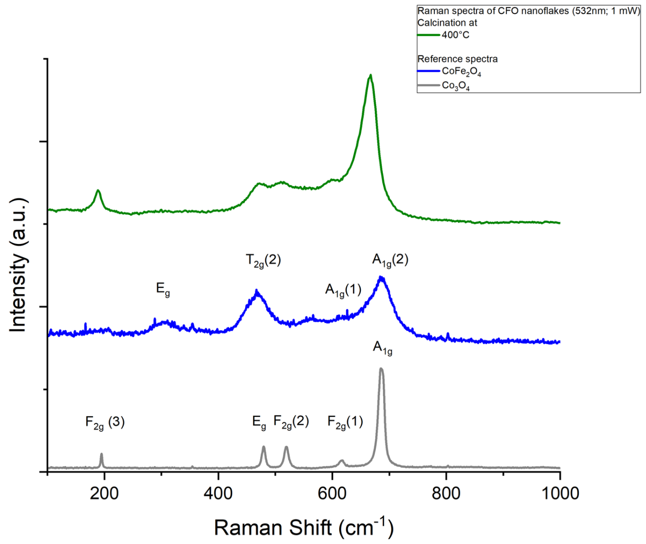

b)

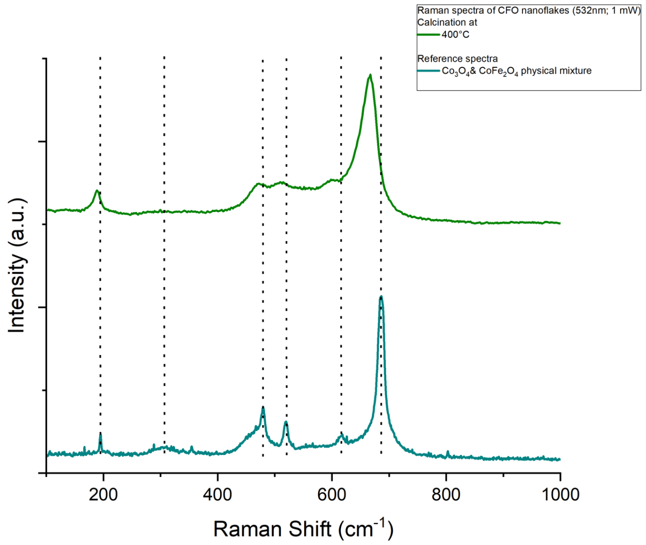

c)

Figure S15: a) Nanoflakes calcined at different temperatures compared to the reference materials Co_3O_4 and $CoFe_2O_4$. b) Figure S15b. Nanoflakes calcined at 400 °C compared to the reference materials Co_3O_4 and $CoFe_2O_4$. c) Figure S15c. Nanoflakes calcined at 400 °C compared to a mixture of the reference materials Co_3O_4 and $CoFe_2O_4$ (calculated from the individual spectra of the two components).

After applying sequentially increasingly higher laser powers followed by recording spectra at 1 mW at the same spot (as described by Rivas-Murias et al. in [12]), the materials calcined at 400 °C (Fig S16) and 900 °C (Fig S17) reveal spectra that evolve into a spectrum that is characteristic for CFO, indicated by the disappearance of the $F_{2g}(3)$ band characteristic for Co_3O_4 and Co_2FeO_4 and the appearance of the E_g band and $A_{1g}(1)$ band typical for CFO. It should be noted that this state is reached at a lower laser power (25 mW compared to 50mW) when the material is calcined at a higher temperature (900 °C compared to 400 °C), which might be caused by the fact that the sample calcined at higher temperature is more homogeneous and changes are less prominent. An additional Co containing phase could not be detected and the signal to noise ratio of the spectra indicates a poor crystallinity of the resulting material which may contain a high concentration of defects induced by the high laser power.

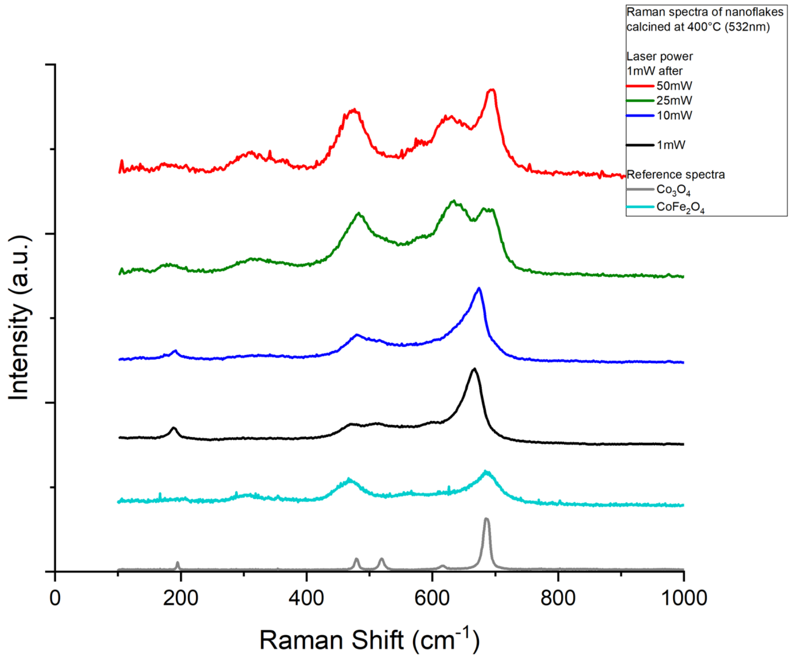

Figure S 16. Nanoflakes calcined at 400°C treated with sequentially increasing laser power compared to the reference materials Co_3O_4 and $CoFe_2O_4$.

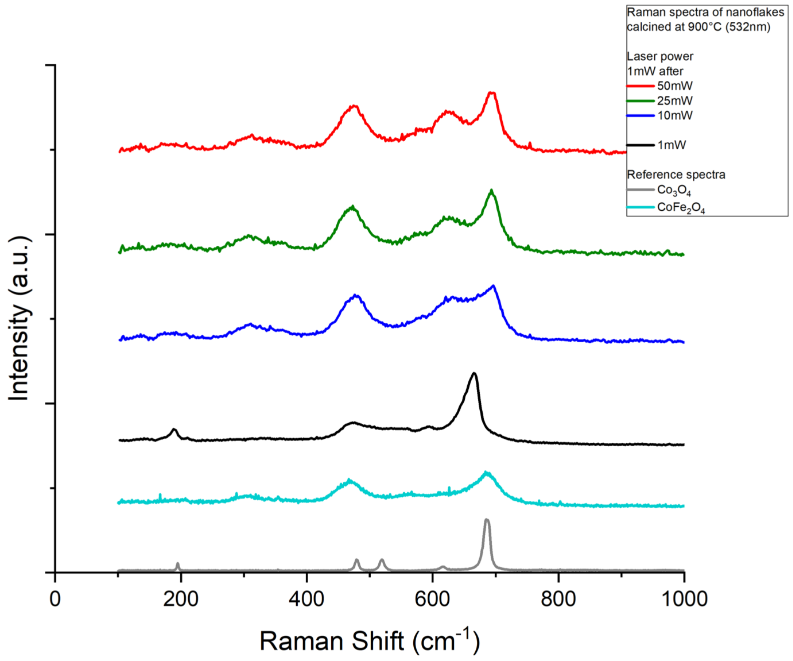

Figure S 17: Nanoflakes calcined at 900°C treated with sequentially increasing laser power compared to the reference materials Co_3O_4 and $CoFe_2O_4$.

X-ray absorption spectroscopy

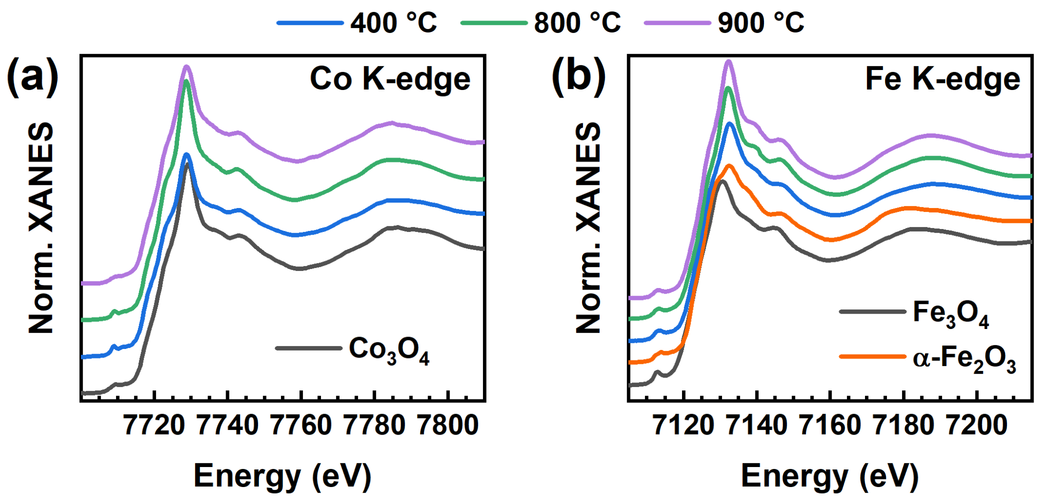

Figure S 18. The shape and position of the (a) XANES profile at Co K-edge shows very good agreement to the profiles of Co_3O_4 (black). In contrast, the shape of the (b) XANES profiles recorded at the Fe K-edge agrees well with the one of Fe_3O_4 (black), while its position is shifted to higher energies such as the α-Fe_2O_3 reference (orange) indicating a higher Fe oxidation state in a spinel structure. The reference spectra are extracted from [18].

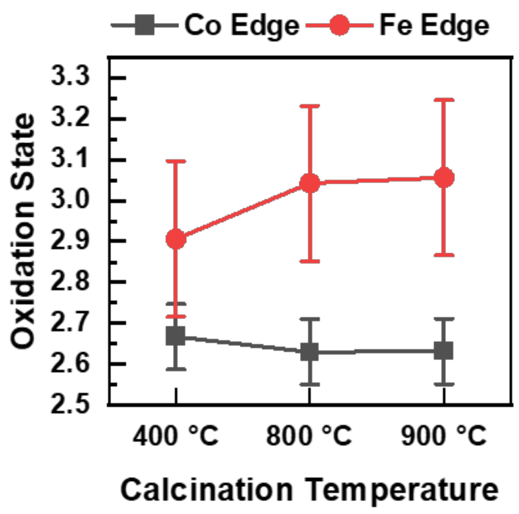

Figure S 19: The trends in oxidation state for different calcination temperatures. Average oxidation states and their uncertainties are obtained from the edge position and linear calibration curves deduced from the edge positions of reference compounds (Table S 5).

The metal oxidation states have been estimated using the edge shift of the metal K-edges with formal oxidation state as determined from reference compounds. The edge position of the metal K-edges has been determined using the integral method as described elsewhere.[18]

For the 400 °C sample the average oxidation state of cobalt is 2.7±0.1 and for iron 2.9±0.2. The oxidation state of cobalt is slightly higher than 2.5, the nominally expected for Co_2FeO_4,

while the oxidation state of iron is 3+ within error bars. All other characterization methods showed the presence of two spinel phases with different compositions.

After calcination at 800 °C the oxidation state of cobalt is somewhat decreased with 2.6±0.1 and the oxidation state of iron is 3.0±0.2 while for the sample calcined at 900 °C the oxidation states are 2.6±0.1 for cobalt and 3.1±0.2 for iron.

Table S 5: Oxidation state and absorption edge position in eV for CoO_X and FeO_X reference compounds and the samples calcined at different temperatures. Edge positions of reference compounds extracted from [18]

**Co K-edge**	**#**	**Oxidation State**	**Edge Position (eV)**
**References**	***rs*-CoO**	2	7718.22
	***w*-CoO**	2	7718.41
	**β-$Co(OH)_2$**	2	7718.47
	**Co_3O_4 (Sigma Aldrich)**	2.67	7720.03
	**CoOOH**	3.0	7721.03
	**$Na_{0.77}CoO_2$**	3.23	7721.37
	**$Na_{0.59}CoO_2$**	3.41	7721.93
**Samples**	**400 °C**	2.67 ± 0.08	7720.05
	**800 °C**	2.63 ± 0.08	7719.96
	**900 °C**	2.63 ± 0.08	7719.96

**Fe K-edge**	**#**	**Oxidation State**	**Edge Position (eV)**
**References**	**FeO**	2	7120.37
	**Fe_3O_4**	2.67	7122.34
	**α-Fe_2O_3**	3	7123.54
	**FeOOH**	3	7123.71
**Samples**	**400 °C**	2.91 ± 0.19	7123.27
	**800 °C**	3.04 ± 0.19	7123.71
	**900 °C**	3.06 ± 0.19	7123.76

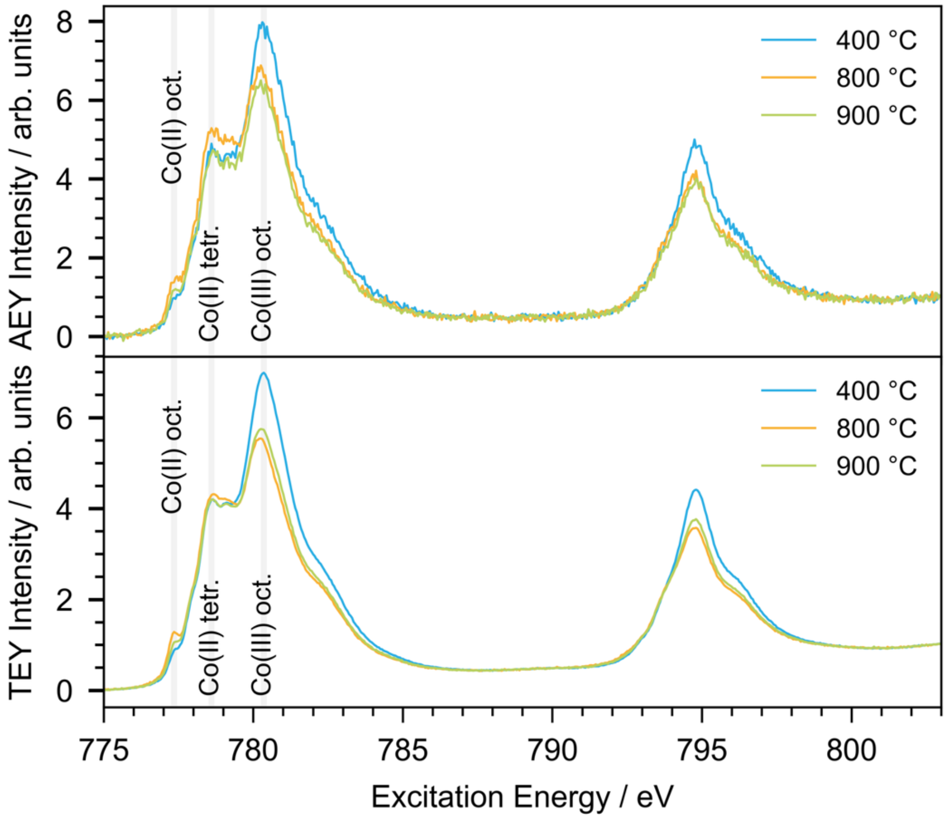

Figure S 20: Cobalt L-edge absorption spectra in surface-sensitive Auger electron yield (AEY, top) and total electron yield (TEY, bottom, sampling more of the subsurface) for three samples calcined at 400 °C, 800 °C and 900 °C. The samples feature signals from octahedrally coordinated Co(**II**) at 777.3 eV,[29] which is not present in pure Co_3O_4.

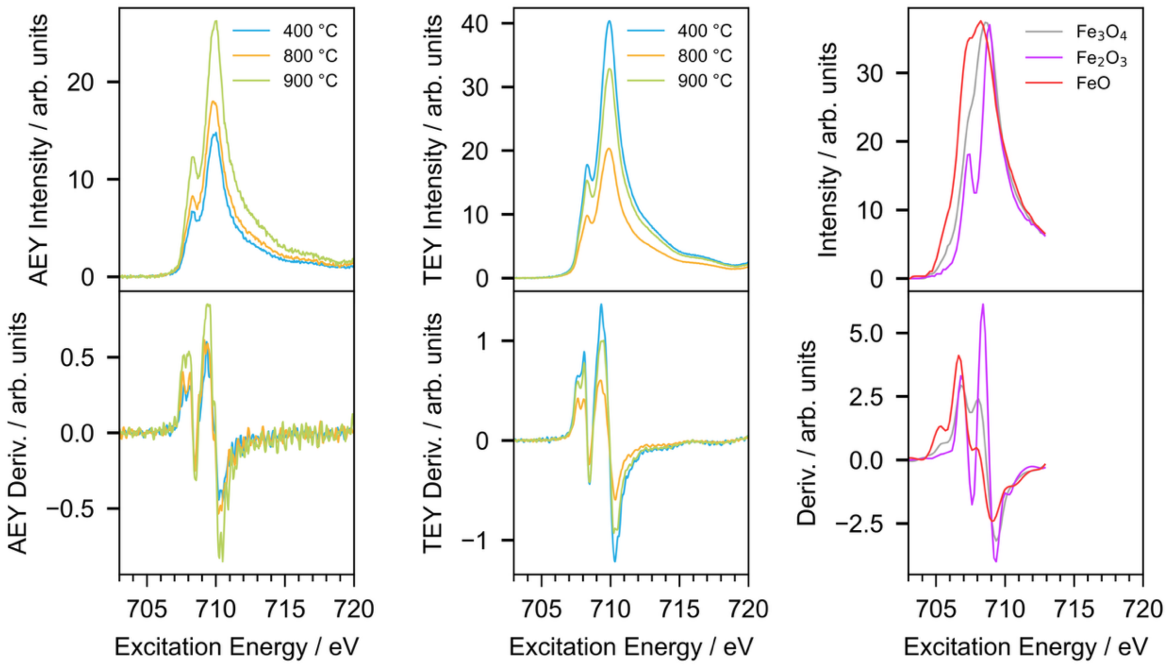

Figure S 21: Iron L_3-edges in AEY(left column), TEY (middle column) and for reference oxides FeO, Fe_2O_3 and Fe_3O_4 (digitized from ref. [30], right column). The measured spectra for all samples closely resemble the pure Fe(III) state as they do not feature the low-energy shoulder present in Fe(II)-containing specimens (see FeO and Fe_3O_4). The lower panels show the first derivative of the data to enhance the visibility of potential low-energy shoulders in the data.

References

[1] A. Rabe, J. Büker, S. Salamon, A. Koul, U. Hagemann, J. Landers, K. Friedel Ortega, B. Peng, M. Muhler, H. Wende, *Chem. - Eur. J.* **2021**, *27*, 17038-17048.

[2] S. L. Thomae, N. Prinz, T. Hartmann, M. Teck, S. Correll, M. Zobel, *Rev. Sci. Instrum.* **2019**, *90*, 043905.

[3] X. Yang, P. Juhas, C. L. Farrow, S. J. Billinge, *arXiv preprint arXiv:1402.3163* **2014**.

[4] C. Farrow, P. Juhas, J. Liu, D. Bryndin, E. Božin, J. Bloch, T. Proffen, S. Billinge, *J. Phys.: Condens. Matter* **2007**, *19*, 335219.

[5] A. Buades, B. Coll, J.-M. Morel, in *2005 IEEE computer society conference on computer vision and pattern recognition (CVPR'05), Vol. 2*, Ieee, **2005**, pp. 60-65.

[6] K. Dabov, A. Foi, V. Katkovnik, K. Egiazarian, in *Image Processing: Algorithms and Systems VI, Vol. 6812*, SPIE, **2008**, pp. 62-73.

[7] B. Ravel, M. Newville, *J. Synchrotron Radiat.* **2005**, *12*, 537-541.

[8] aM. Newville, *J. Synchrotron Radiat.* **2001**, *8*, 322-324; bJ. Timoshenko, A. Anspoks, A. Kalinko, A. Kuzmin, *physica status solidi (a)* **2015**, *212*, 265-273.

[9] aG. Kresse, J. Furthmüller, *Phys. Rev. B* **1996**, *54*, 11169; bG. Kresse, J. Furthmüller, *Computational materials science* **1996**, *6*, 15-50.

[10] aJ. KresseG, *PhysRevB* **1999**, *59*, 1758G1775; bP. E. Blöchl, *Phys. Rev. B* **1994**, *50*, 17953.

[11] S. L. Dudarev, G. A. Botton, S. Y. Savrasov, C. Humphreys, A. P. Sutton, *Phys. Rev. B* **1998**, *57*, 1505.

[12] aB. Rivas-Murias, M. Testa-Anta, A. S. Skorikov, M. Comesaña-Hermo, S. Bals, V. Salgueiriño, *Nano Letters* **2023**, *23*, 1688-1695; bB. Rivas-Murias, V. Salgueiriño, *Journal of Raman Spectroscopy* **2017**, *48*, 837-841.

[13] O. Bulavchenko, O. Venediktova, T. Afonasenko, P. Tsyrul'Nikov, A. Saraev, V. Kaichev, S. Tsybulya, *RSC Adv.* **2018**, *8*, 11598-11607.

[14] J. P. Perdew, A. Ruzsinszky, G. I. Csonka, O. A. Vydrov, G. E. Scuseria, L. A. Constantin, X. Zhou, K. Burke, *arXiv preprint arXiv:0707.2088* **2007**.

[15] aS. Hirai, W. L. Mao, *Appl. Phys. Lett.* **2013**, *102*, 041912; bP. Smith, C. Spencer, R. Stillwell, *J. Phys. Chem. Solids* **1978**, *39*, 107-111.

[16] L. Chen, Y. Yang, X. Meng, *Appl. Phys. Lett.* **2013**, *102*, 203102.

[17] aS. Regmi, Z. Li, S. KC, R. Mahat, A. Rastogi, R. Datta, A. Gupta, *Appl. Phys. Lett.* **2022**, *121*, 102401; bY. Qu, H. Yang, N. Yang, Y. Fan, H. Zhu, G. Zou, *Mater Lett* **2006**, *60*, 3548-3552; cL. Shen, M. Althammer, N. Pachauri, B. Loukya, R. Datta, M. Iliev, N. Bao, A. Gupta, *J. Cryst. Growth* **2014**, *390*, 61-66.

[18] F. T. Haase, A. Rabe, F.-P. Schmidt, A. Herzog, H. S. Jeon, W. Frandsen, P. V. Narangoda, I. Spanos, K. Friedel Ortega, J. Timoshenko, *J Am Chem Soc* **2022**, *144*, 12007-12019.

[19] A. Walsh, S.-H. Wei, Y. Yan, M. Al-Jassim, J. A. Turner, M. Woodhouse, B. Parkinson, *Phys. Rev. B* **2007**, *76*, 165119.

[20] Z. Li, E. Fisher, J. Liu, M. Nevitt, *J. Mater. Sci.* **1991**, *26*, 2621-2624.

[21] L. Bai, M. Pravica, Y. Zhao, C. Park, Y. Meng, S. V. Sinogeikin, G. Shen, *J. Phys.: Condens. Matter* **2012**, *24*, 435401.

[22] F. T. Haase, A. Rabe, F.-P. Schmidt, A. Herzog, H. S. Jeon, W. Frandsen, P. V. Narangoda, I. Spanos, K. Friedel Ortega, J. Timoshenko, T. Lunkenbein, M. Behrens, A. Bergmann, R. Schlögl, B. Roldan Cuenya, *J Am Chem Soc* **2022**, *144*, 12007-12019.

[23] Z.-A. Li, N. Fontaíña-Troitiño, A. Kovács, S. Liébana-Viñas, M. Spasova, R. E. Dunin-Borkowski, M. Müller, D. Doennig, R. Pentcheva, M. Farle, *Sci. Rep.* **2015**, *5*, 1-6.

[24] aS. A. McEnroe, B. Carter-Stiglitz, R. J. Harrison, P. Robinson, K. Fabian, C. McCammon, *Nat. Nanotechnol.* **2008**, *3*, 58-58; bR. Pentcheva, H. S. Nabi, *Phys. Rev. B* **2008**, *77*, 172405.

[25] T. Daou, J. Grenèche, G. Pourroy, S. Buathong, A. Derory, C. Ulhaq-Bouillet, B. Donnio, D. Guillon, S. Begin-Colin, *Chem Mater* **2008**, *20*, 5869-5875.

[26] M. TAKAHASHI, M. E. FINE, *J. Am. Ceram. Soc.* **1970**, *53*, 633-634.

[27] K. J. Kim, H. K. Kim, Y. R. Park, G. Y. Ahn, C. S. Kim, J. Y. Park, *Hyperfine interactions* **2006**, *169*, 1363-1369.

[28] T. M. C. Dinh, A. Barnabe, M. A. Bui, C. Josse, T. Hungria, C. Bonningue, L. Presmanes, P. Tailhades, *Crystengcomm* **2018**, *20*, 6146-6155.

[29] A. Bergmann, T. E. Jones, E. Martinez Moreno, D. Teschner, P. Chernev, M. Gliech, T. Reier, H. Dau, P. Strasser, *Nat. Catal.* **2018**, *1*, 711-719.

[30] C. Chen, C. Dong, S. Rao, G. Chern, M. Chen, M. Wu, C. Chang, *J. Phys.: Condens. Matter* **2008**, *20*, 255236.
